# The Intestinal Transporter SLC30A1 Plays a Critical Role in Regulating Systemic Zinc Homeostasis

**DOI:** 10.1002/advs.202406421

**Published:** 2024-10-18

**Authors:** Shumin Sun, Enjun Xie, Shan Xu, Suyu Ji, Shufen Wang, Jie Shen, Rong Wang, Xinyi Shen, Yunxing Su, Zijun Song, Xiaotian Wu, Jiahui Zhou, Zhaoxian Cai, Xiaopeng Li, Yan Zhang, Junxia Min, Fudi Wang

**Affiliations:** ^1^ The First Affiliated Hospital Institute of Translational Medicine Zhejiang Key Laboratory of Frontier Medical Research on Cancer Metabolism Zhejiang University School of Medicine Hangzhou 310058 China; ^2^ The Second Affiliated Hospital School of Public Health Zhejiang University School of Medicine Hangzhou 310058 China; ^3^ Department of Biophysics and Department of Pathology Sir Run Run Shaw Hospital Zhejiang University School of Medicine Hangzhou 310016 China; ^4^ The First Affiliated Hospital Basic Medical Sciences School of Public Health Hengyang Medical School University of South China Hengyang 421001 China

**Keywords:** intestinal barrier integrity, ion selectivity, SLC30A1 structure, zinc homeostasis

## Abstract

The essential trace element, zinc, regulates virtually all aspects of cellular physiology, particularly cell proliferation and survival. Diverse families of metal transporters, metallothioneins, and metal‐responsive transcriptional regulators are linked to zinc homeostasis. However, the mechanism underlying the regulation of systemic zinc homeostasis remains largely unknown. Here, it is reported that the intestinal transporter SLC30A1 plays an essential role in maintaining systemic zinc homeostasis. Using several lines of tissue‐specific knockout mice, it is found that intestinal Slc30a1 plays a critical role in survival. Furthermore, lineage tracing reveals that Slc30a1 is localized to the basolateral membrane of intestinal epithelial cells (IECs). It is also found that Slc30a1 safeguards both intestinal barrier integrity and systemic zinc homeostasis. Finally, an integrative analysis of the cryo‐EM structure and site‐specific mutagenesis of human SLC30A1 are performed and a zinc transport mechanism of SLC30A1 unique within the SLC30A family, with His43 serving as a critical residue for zinc selectivity, is identified.

## Graphical abstract

Intestinal SLC30A1 plays an essential role for controlling systemic zinc homeostasis via its basolateral membrane‐localized transporter activity of zinc ions from intestinal epithelial cells (IECs) to the circulation. The lethal phenotype of intestinal *Slc30a1* deficient mice can be fully rescued by systemic zinc supplementation. The Cryo‐EM structure and mutagenesis of human SLC30A1 reveal the protein's mechanism for transporting metal ions.

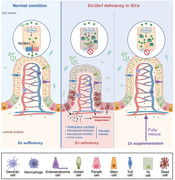

## 1 Introduction

As an essential trace element, zinc regulates a wide range of cellular processes, including cell proliferation, DNA synthesis, gene transcription, and protein synthesis. According to the World Health Organization,^[^ ^1^ ^]^ zinc deficiency is a global public health problem, causing diverse symptoms such as impaired growth and/or development, immune system disorders, and impaired neurological function.^[^ ^2^, ^3^ ^]^ Zinc homeostasis is therefore critical for maintaining health. A wide range of protein families have been reported to regulate zinc levels, including metal transporters, metallothioneins, and metal‐responsive transcription factors. The majority of systemic zinc ions are intracellular,^[^ ^4^ ^]^ either bound to proteins/enzymes or existing as free ions. The compartmentalized distribution of zinc in various organelles is tightly regulated by two families of metal transporters, namely the SLC30A (also known as ZnT) and SLC39A (also known as Zrt‐ and Irt‐like proteins, or ZIP) families. The SLC39A protein family contains 14 members (SLC39A1 through SLC39A14) that transport zinc ions from the extracellular space and organelles into the cytoplasm, while the SLC30A protein family contains ten members (SLC30A1 through SLC30A10) that transport zinc ions from the cytoplasm into the organelles and the extracellular space.^[^ ^5^, ^6^ ^]^

Their distinct localization to either the plasma membrane or organelle membranes endows specific functions to these zinc transporters. In recent decades, increasing attention has been paid to the various diseases associated with the altered function of zinc transporters,^[^ ^7^, ^8^, ^9^, ^10^, ^11^, ^12^, ^13^, ^14^, ^15^, ^16^, ^17^, ^18^, ^19^ ^]^ primarily via altered zinc homeostasis. E.g., a mutation in *SLC39A4* has been linked to the zinc metabolism disorder acrodermatitis enteropathica.^[^ ^20^, ^21^ ^]^ Moreover, we found that zinc influx via SLC39A5 in pancreatic β cells is required for insulin secretion,^[^ ^22^ ^]^ and we showed that SLC39A10 is essential for maintaining both hematopoiesis^[^ ^23^ ^]^ and p53‐mediated macrophage survival.^[^ ^24^ ^]^

Zinc absorption in the intestine regulates systemic zinc homeostasis, and we previously identified SLC39A4 as a zinc transporter localized to the apical membrane, where it mediates the uptake of zinc ions from the intestinal lumen.^[^ ^25^, ^26^ ^]^ However, the precise mechanism by which zinc ions are transported into the circulation in order to maintain systemic zinc homeostasis remains poorly understood.

The mammalian *SLC30A1* gene was cloned in 1995^[^ ^27^ ^]^ and subsequently found to be expressed ubiquitously in the plasma membrane.^[^ ^28^ ^]^ Since it was cloned, Slc30a1 has been extensively investigated for its role in regulating zinc homeostasis in vitro.^[^ ^29^ ^]^ The expression of Slc30a1 is largely regulated by dietary zinc^[^ ^30^ ^]^ and also mediated by cellular zinc levels.^[^ ^31^ ^]^ While most of the previous studies on Slc30a1 were performed in vitro, its physiological essential role in vivo was reported in 2004 that showed *Slc30a1* knockout mice are embryonic lethal, suggesting that this transporter plays an essential role in early development.^[^ ^32^ ^]^ However, due to the embryonic lethality in global *Slc30a1* knockout mice, the tissue‐specific functions of SLC30A1 have not been well investigated.

Moreover, SLC30A1 expression and gene mutations have been associated with the development and/or progression of several tumor types,^[^ ^33^, ^34^ ^]^ which indicates that its disrupted function could lead to pathological conditions. To address these questions, we generated and functionally characterized an inducible global *Slc30a1* knockout mouse, tissue‐specific *Slc30a1* knockout mice, and lineage tracing mice, and we analyzed the cryo‐EM structure of wild‐type and mutated forms of human SLC30A1. We found that intestinal SLC30A1 is essential for embryonic survival and plays a critical role in regulating systemic zinc homeostasis. In addition, we resolved the cryo‐EM structure of human SLC30A1 at 3.7‐Å resolution, and gained novel mechanistic insights into the structure‐function relationship of SLC30A1 and its selectivity for transporting zinc ions.

## 2 Results

### 2.1 Intestinal *Slc30a1* Plays an Essential Role in Mice

**Figure 1:**
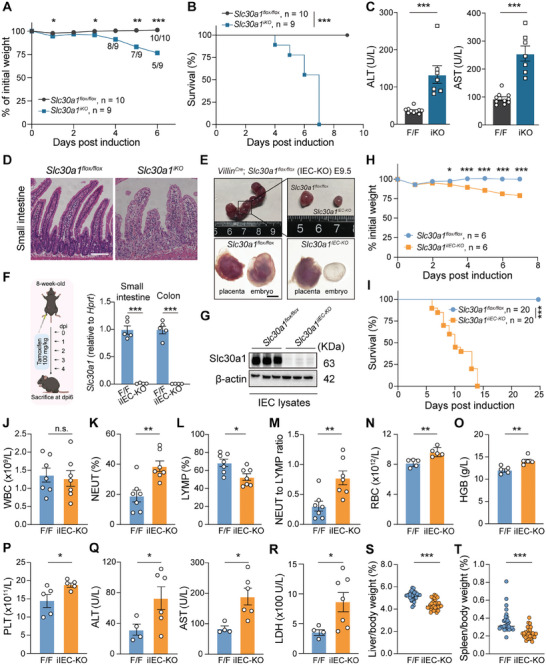
Acute loss of intestinal Slc30a1 induces rapid mortality in mice. A) Time course of relative body weight measured in the indicated mice after tamoxifen induction. n = 9–10 mice per group. B) Survival curve of adult control (*Slc30a1^flox/flox^*) and *Slc30a1^iKO^* mice following tamoxifen injections to globally knock out *Slc30a1* expression in the *Slc30a1^iKO^* mice. n = 9–10 mice per group. C) Summary of serum alanine transaminase (ALT) and aspartate aminotransferase (AST) levels in *Slc30a1^flox/flox^* and *Slc30a1^iKO^* mice at dpi6. n = 7–10 mice per group. D) Representative images of small intestine sections obtained from control *Slc30a1^flox/flox^* and *Slc30a1^iKO^* mice at day 6 post‐induction (dpi6) and stained with H&E. E) Representative images of embryos obtained from heterozygous parents at embryonic day 9.5 (E9.5) (upper images), and magnified images of *Slc30a1^flox/flox^* and *Slc30a1^IEC‐KO^* E9.5 embryos (lower images). F) Protocol for inducing *Slc30a1* knockout by injecting tamoxifen for five consecutive days (100 mg k^−1^g body weight/day, with dpi0 defined as the first day of tamoxifen injections) in control and *Slc30a1^iIEC‐KO^* mice (left), and summary of *Slc30a1* mRNA measured in the small intestine and colon (right). n = 4–5 mice per group. G) Western blot analysis of Slc30a1 protein in IECs obtained from *Slc30a1^flox/flox^* and *Slc30a1^iIEC‐KO^* mice at dpi6; β‐actin was included as a loading control. H) Time course of the relative body weight of the indicated mice following tamoxifen induction. n = 6 mice per group. I) Survival curve of the indicated mice following tamoxifen induction. n = 20 mice per group. J–M) Total white blood cell (WBC) count (J), percentages of neutrophils (K) and lymphocytes (L), and the neutrophil‐to‐lymphocyte ratio (M) measured in the indicated mice at dpi6. n = 7 mice per group. N–P) Red blood cell (RBC) count (N), hemoglobin concentration (O), and platelet count (P) measured in the indicated mice at dpi6. n = 5 mice per group. Q) Summary of serum ALT and AST levels measured in the indicated mice at dpi6. n = 4–6 mice per group. R) Summary of serum LDH levels measured in the indicated mice at dpi6. n = 4–7 mice per group. S,T) Summary of relative liver (S) and spleen (T) weight measured in the indicated mice at dpi6. n = 30–31 mice per group. In this and subsequent figures, results are representative of at least two independent experiments; in addition, unless indicated otherwise all summary data are presented as the mean ± SEM, and each symbol represents one mouse. *p < 0.05, **p < 0.01, ***p < 0.001, and n.s., not significant; Student's *t‐*test (A, C, H, and J–T) or log‐rank (Mantel‐Cox) test (B and I). Scale bars, 100 µm (D) and 2000 µm (E). See also Figures S1–S3 (Supporting Information).

First, we generated a UBC‐CreERT–driven inducible *Slc30a1* global knockout mouse line (*Slc30a1^iKO^*) using the Cre‐loxP system (Figure S1A,B, Supporting Information) and found that these mice lost weight rapidly and died within a week of induction (**Figure** **1**A,B), with evidence of tissue damage in the intestine and liver (Figures 1C,D; S1C, Supporting Information). To study the tissue‐specific role of Slc30a1, we then generated intestinal epithelial cell (IEC)‒specific *Slc30a1* knockout (*Slc30a1^IEC‐KO^*) mice and liver‐specific *Slc30a1* knockout (*Slc30a1^LKO^*) mice (Figure S2A, Supporting Information). Surprisingly, we found that the *Slc30a1^IEC‐KO^* mice are embryonic lethal (Figure 1E), whereas the liver‐specific *Slc30a1* knockout mice are viable (Figure S2B, Supporting Information).

Next, we generated a Villin‐CreERT2–driven inducible IEC‐specific *Slc30a1* knockout mouse line (*Slc30a1^iIEC‐KO^*) (Figure S2A, Supporting Information) to examine the functional role of Slc30a1 in the intestine; knockout in IECs was induced by injecting the *Slc30a1^iIEC‐KO^* mice with tamoxifen (Figure 1F). We first detected the extensive but distinguished expression of Slc30a1 protein throughout the small intestine, while higher expression was found in the proximal portion (Figure S2C, Supporting Information). And we confirmed the near‐complete loss of *Slc30a1* mRNA (Figure 1F) and Slc30a1 protein (Figure 1G; Figure S2D, Supporting Information) in both the small intestine and colon of adult *Slc30a1^iIEC‐KO^* mice 6 days post‐induction (dpi6); unless indicated otherwise, all subsequent results were obtained from adult *Slc30a1^iIEC‐KO^* mice at dpi6, and *Slc30a1^flox/flox^* littermates were used as controls. We found that starting at dpi3, the *Slc30a1^iIEC‐KO^* mice began to experience significant weight loss and died shortly thereafter, with 100% mortality by dpi14 (Figure 1H,I; Figure S2E–J, Supporting Information). These results indicate that intestinal Slc30a1 plays an essential role in survival, well into adulthood. Although the total white blood cell count was similar between *Slc30a1^iIEC‐KO^* mice and controls (Figure 1J), *Slc30a1^iIEC‐KO^* mice had a significantly higher percentage of neutrophils (Figure 1K) and a significantly lower percentage of lymphocytes (Figure 1L), resulting in an increased neutrophil‐to‐lymphocyte ratio (Figure 1M) and suggesting activation of an inflammatory response.^[^ ^35^ ^]^ Moreover, the *Slc30a1^iIEC‐KO^* mice had a significantly higher red blood cell count, hemoglobin concentration, and platelet count compared to controls (Figure 1N–P), which may be related to the activation of inflammatory responses.^[^ ^36^ ^]^ Finally, the *Slc30a1^iIEC‐KO^* mice had significantly higher serum ALT (alanine transaminase), AST (aspartate aminotransferase), and LDH (lactate dehydrogenase) levels (Figure 1Q,R), reduced liver and spleen mass (Figure 1S,T), and histopathological changes in the liver and spleen (Figure S2K, Supporting Information), indicating damage in these organs.

Interestingly, by analyzing human genome‐wide association study (GWAS) data obtained from the UK Biobank (Figure S3A, Supporting Information), we found a significant correlation (*p* = 9.39 × 10^−5^) between an intergenic mutation located between the *RD3* and *SLC30A1* genes on chromosome 1 (n.211740959 C > T; Figure S3B, Supporting Information) and Crohn's disease in male patients (based on 471 cases and 16,6517 controls). Combined with the phenotypes we observed in *Slc30a1^iIEC‐KO^* mice, these findings provided strong rationale for further study of the critical physiological and pathological roles of SLC30A1 in the intestine.

### 2.2 Loss of Slc30a1 Leads to Severe Intestinal Damage

**Figure 2:**
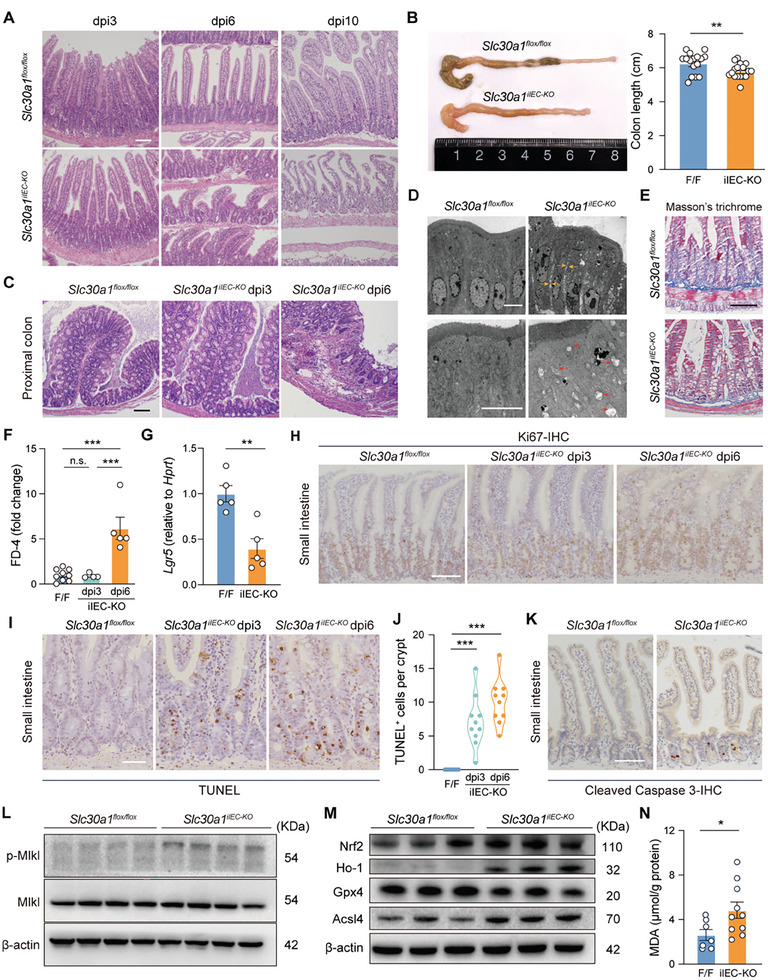
Loss of intestinal Slc30a1 disrupts gut integrity and triggers cell death. A) Representative images of Swiss rolls prepared from small intestine samples obtained from *Slc30a1^flox/flox^* and *Slc30a1^iIEC‐KO^* mice at dpi3, dpi6, and dpi10, sectioned, and stained with H&E. B) Representative picture of the colon (left) and summary of colon length (right) in *Slc30a1^flox/flox^* and *Slc30a1^iIEC‐KO^* mice at dpi6. n = 16–17 mice per group. C) Representative images of proximal colon sections obtained from *Slc30a1^flox/flox^* and *Slc30a1^iIEC‐KO^* mice at dpi3 and dpi6 and stained with H&E. D) Representative transmission electron microscopy images of small intestine sections at dpi6. The yellow arrows in the upper‐right panel indicate increased intercellular space in the *Slc30a1^iIEC‐KO^* mice, and the red arrows in the lower‐right panel indicate abnormal mitochondria in the *Slc30a1^iIEC‐KO^* mice. E) Representative images of small intestine sections obtained from *Slc30a1^flox/flox^* and *Slc30a1^iIEC‐KO^* mice at dpi6 and stained with Masson's trichrome. F) Summary of intestinal permeability in the indicated mice measured using the 4‐kDa fluorescent dextran‐FITC (FD‐4) test, expressed relative to control. n = 4–9 mice per group. G) Summary of *Lgr5* mRNA measured in IECs obtained from *Slc30a1^flox/flox^* and *Slc30a1^iIEC‐KO^* mice at dpi6. n = 5 mice per group. H) Representative images of small intestine sections obtained from *Slc30a1^flox/flox^* and *Slc30a1^iIEC‐KO^* mice at dpi3 and dpi6, and stained for Ki67 to label proliferating cells. I,J) Representative images of TUNEL‐stained small intestinal sections obtained from the indicated mice (I) and summary of TUNEL‐positive cells per crypt (J) (n = 10 crypts pooled from 3 biological replicates per group). K) Representative images of small intestinal sections prepared from *Slc30a1^flox/flox^* and *Slc30a1^iIEC‐KO^* mice at dpi6 and stained for cleaved Caspase 3 to label apoptotic cells. L) Western blot analysis of total Mlkl and phosphorylated Mlkl (p‐Mlkl) in IECs obtained from the indicated mice at dpi6; β‐actin was included as a loading control. M) Western blot analysis of Nrf2, Ho‐1, Gpx4, and Acsl4 in IECs obtained from the indicated mice at dpi6; β‐actin was included as a loading control. N) Summary of malondialdehyde content in IECs obtained from the indicated mice at dpi6. n = 7–10 mice per group. *p < 0.05, **p < 0.01, ***p < 0.001, and n.s., not significant; Student's *t‐*test (B, G, and N) or one‐way ANOVA with Tukey's post hoc test (F and J). Scale bars, 100 µm (A, C, E, H, and K), 5 µm (D), and 50 µm (I). See also Figures S4 and S5 (Supporting Information).

Hematoxylin and eosin (H&E) staining of Swiss rolls prepared from small intestine samples showed a disrupted epithelial structure in *Slc30a1^iIEC‐KO^* mice that was visible at dpi6 and was more severe by dpi10, particularly in the crypt region (**Figure** **2**A). In addition, the colon was significantly shorter (Figure 2B) compared to control mice and had an altered structure at dpi6 in the *Slc30a1^iIEC‐KO^* mice (Figure 2C), suggesting the activation of intestinal inflammation. Moreover, compared to controls the IECs in *Slc30a1^iIEC‐KO^* mice had severely disrupted cell‐cell gap junctions (Figure 2D, upper panel, yellow arrows), an enlarged intercellular space, and damaged mitochondria (Figure 2D, lower panel, red arrows) based on transmission electron microscopy; finally, Masson's trichrome staining revealed reduced extracellular matrix (Figure 2E). Consistent with these morphological and cellular changes, a fluorescein isothiocyanate—dextran (FD‐4) uptake assay revealed increased intestinal permeability in *Slc30a1^iIEC‐KO^* mice at dpi6 compared to control mice (Figure 2F).

Next, given that the intestine contains a wide range of cell types (Figure S4A, Supporting Information), we analyzed whether the loss of intestinal Slc30a1 causes a change in any of these cell types. We found decreased expression of the stem cell marker gene *Lgr5* (Figure 2G) and increased protein levels of the Paneth cell marker Lyz1 (Figure S4B, Supporting Information)—but no change in goblet cells based on periodic acid–Schiff (PAS) staining (Figure S4C,D, Supporting Information)—in *Slc30a1^iIEC‐KO^* mice compared to control mice. Moreover, we observed more Ki67‐positive (i.e., proliferating) TA (transit amplifying) cells (Figure 2H) in *Slc30a1^iIEC‐KO^* mice, but reduced BrdU incorporation in crypt cells (Figure S4E, Supporting Information), suggesting cell cycle arrest. In addition, TUNEL staining showed significantly more dead cells per crypt in *Slc30a1^iIEC‐KO^* mice at dpi3 compared to controls (despite no significant change in intestinal permeability at this time point), with even more dead cells present at dpi6 (Figure 2I,J). Given these results, we conclude that loss of intestinal Slc30a1 induces cell death in crypts, leading to impaired intestinal integrity. Notably, we observed the co‐occurrence of several forms of regulated cell death in *Slc30a1^iIEC‐KO^* mice. In IECs of *Slc30a1^iIEC‐KO^* mice, we observed the upregulated cleaved Caspase 3, the main executioner of apoptosis,^[^ ^37^ ^]^ indicating activation of apoptosis (Figure 2K), and the increased phosphorylation of the pseudokinase mixed lineage kinase domain‐like protein (Mlkl) (Figure 2L), whose oligomerization and translocation to the plasma membrane as an indicator of necroptosis.^[^ ^38^, ^39^ ^]^ In addition, we found the increased long‐chain fatty acid‐CoA 4 (Acsl4) (Figure 2M; Figure S4F–I, Supporting Information) that has been reported to accelerate the production of pro‐ferroptotic lipid substrates,^[^ ^40^ ^]^ and the decreased glutathione peroxidase 4 (Gpx4) (Figure 2M; Figure S4F–I, Supporting Information) that could result in attenuated antioxidative responses,^[^ ^41^ ^]^ as well as the accumulated MDA (malondialdehyde) (Figure 2N), a byproduct of lipid peroxidation,^[^ ^42^ ^]^ suggesting the activation of ferroptosis in IECs of *Slc30a1^iIEC‐KO^* mice. However, our attempts to inhibit cell death in IECs in *Slc30a1^iIEC‐KO^* mice using either genetic or pharmacological interventions had only a marginal effect on survival (Figure S5, Supporting Information).

### 2.3 Intestinal *Slc30a1* Knockout Mice have an Overactivated Inflammatory Response

**Figure 3:**
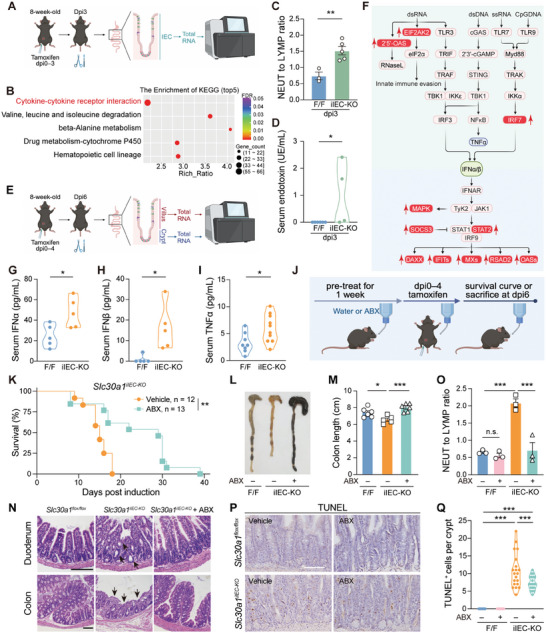
*Slc30a1^iIEC‐KO^* mice have an increased inflammatory response. A) Schematic diagram depicting the strategy for performing RNA‐seq on mouse IECs obtained at dpi3.B) The top 5 KEGG pathways enriched following tamoxifen induction based on FDR (false discovery rate). C,D) Summary of the neutrophil‐to‐lymphocyte ratio (C) and serum endotoxin levels (D) measured in the indicated mice at dpi3. n = 3–6 mice per group. E) Schematic diagram depicting the strategy for performing RNA‐seq on villus and crypt cells obtained at dpi6. F) Map of the representative signal pathways involved in type I interferon production and transduction; the genes labeled in red boxes are significantly upregulated in *Slc30a1^iIEC‐KO^* mice at dpi6. G–I) Summary of the indicated serum cytokines measured in the indicated mice at dpi6. n = 5–10 mice per group. J) Schematic diagram depicting the strategy for treating mice with a cocktail of antibiotics (ABX) or vehicle (ddH_2_O) followed by tamoxifen induction. K) Survival curve of *Slc30a1^iIEC‐KO^* mice treated with ABX or vehicle. n = 12–13 mice per group. L,M) Representative pictures of the colon (L) and summary of colon length (M) in the indicated mice at dpi6. n = 5–6 mice per group. N) Representative images of duodenum and colon sections obtained from the indicated mice at dpi6 and stained with H&E. The arrows in the middle panel indicate the disrupted intestinal structures. O) Summary of the neutrophil‐to‐lymphocyte ratio in the indicated mice at dpi6. n = 3 mice per group. P and Q) Representative images of TUNEL‐stained small intestinal sections obtained from the indicated mice at dpi6 (P) and summary of TUNEL‐positive cells per crypt (Q) (n = 20 crypts pooled from 3 biological replicates per group). *p < 0.05, **p < 0.01, and ***p < 0.001; Student's *t‐*test (C and G–I), Mann‐Whitney *U* test (D), log‐rank (Mantel‐Cox) test (K), or one‐way (M) or two‐way (O and Q) ANOVA with Tukey's post hoc test. Scale bars, 100 µm. See also Figures S6 and S7 (Supporting Information).

To examine the mechanism by which the acute loss of intestinal Slc30a1 rapidly causes severe morbidity and high mortality, we first extracted total RNA from *Slc30a1^iIEC‐KO^* and control IECs at dpi3 and performed transcriptomics analysis (**Figure** **3**A). We found more than 3 000 differentially expressed genes in the *Slc30a1^iIEC‐KO^* group (Figure S6A, Supporting Information); moreover, KEGG (Kyoto Encyclopedia of Genes and Genomes) analysis revealed more than 100 significantly altered signaling pathways (the top five pathways are shown in Figure 3B), with cytokine‐cytokine receptor interaction being the top changed pathway (Figure 3B). Consistent with this finding, we observed an activated inflammatory response in *Slc30a1^iIEC‐KO^* mice as early as dpi3, indicated by an increase in neutrophils (Figure S6B, Supporting Information), a decrease in lymphocytes (Figure S6C, Supporting Information), and an increased neutrophil‐to‐lymphocyte ratio (Figure 3C). In addition, endotoxins were detectable in the serum of *Slc30a1^iIEC‐KO^* mice—but not control mice—at dpi3 (Figure 3D), possibly due to the death of IECs in the crypt region causing increased intestinal permeability. We also measured increased levels of the cytokine IFNγ (interferon gamma) in *Slc30a1^iIEC‐KO^* mice at dpi3 (Figure S6D, Supporting Information).

Because we observed more dead cells specifically in the crypt region of *Slc30a1^iIEC‐KO^* mice at dpi6, we isolated the crypt and villus compartments at dpi6 and performed transcriptomics analysis (Figure 3E). We found robust activation of infectious response pathways in crypt cells (Figure S6E, Supporting Information), but not in the villi (Figure S6F, Supporting Information); most of the differentially expressed genes regulating the inflammatory response in crypts are involved in signaling pathways involving type I interferons (Figure 3F, significantly upregulated genes are shown in red boxes). Furthermore, we found that the cGAS‐STING (cyclic GMP‐AMP synthase/stimulator of interferon genes) pathway^[^ ^43^ ^]^ was activated in *Slc30a1^iIEC‐KO^* mice (Figure S6G–I, Supporting Information); this pathway plays a critical role in activating the innate immune response against DNA viruses and DNA fragments released from dead cells.^[^ ^44^, ^45^ ^]^ Consistent with this finding, serum levels of IFNα, IFNβ, and TNFα—cytokines stimulated by the STING pathway—were also increased in *Slc30a1^iIEC‐KO^* mice compared to control mice (Figure 3G–I). Interestingly, the cGAS‐STING pathway was activated as early as dpi3 (Figure S6J,K, Supporting Information), suggesting that the death of IECs plays a role in this process. Finally, treating *Slc30a1^iIEC‐KO^* mice with the STING antagonist H‐151^[^ ^46^ ^]^ significantly—albeit modestly—prolonged their survival (Figure S6L, Supporting Information). These results indicate that a strongly activated inflammatory response follows the increase in IEC death induced by the loss of intestinal Slc30a1.

Our finding that the loss of intestinal Slc30a1 reduces the integrity of the gut barrier suggests that invasive pathogens may trigger the observed inflammatory response. We therefore examined whether eliminating gut microbes could improve outcome in *Slc30a1^iIEC‐KO^* mice. We treated *Slc30a1^iIEC‐KO^* mice with either plain water or water containing an antibiotic cocktail (ABX) consisting of 1 g L⁻^1^ ampicillin, 1 g L⁻^1^ neomycin, 1 g L⁻^1^ metronidazole, and 0.5 g L⁻^1^ vancomycin^[^ ^47^ ^]^ (Figure 3J). We found that the ABX‐treated *Slc30a1^iIEC‐KO^* mice survived significantly longer than the untreated *Slc30a1^iIEC‐KO^* mice (Figure 3K), with reduced morphological changes in the intestine (Figure 3L–N), a reduced inflammatory response (Figure 3O; Figure S7A–D, Supporting Information), and reduced death of IECs (Figure 3P,Q), but had no effect on expression of the stem cell marker *Lgr5* (Figure S7E, Supporting Information). Importantly, ABX treatment significantly reduced the production of cytokines involved in the cGAS‐STING pathway (Figure S7F–I, Supporting Information). Taken together, these findings indicate that the death of IECs—and the resulting increase in intestinal permeability—drives activation of the interferon response, ultimately leading to mortality in *Slc30a1^iIEC‐KO^* mice.

### 2.4 Intestinal Slc30a1 Dictates Systemic Zinc Homeostasis

**Figure 4:**
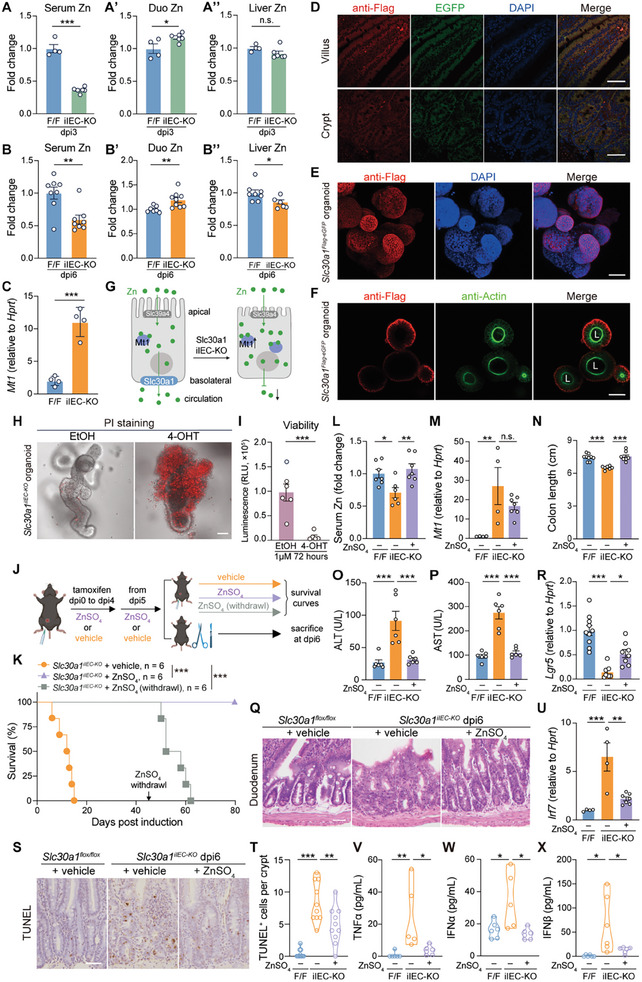
Intestinal Slc30a1 regulates circulating zinc levels. A) Total zinc levels measured using ICP‐MS in the serum (A), duodenum (A’), and liver (A’’) of the indicated mice at dpi3. n = 4–6 mice per group. B) Total zinc levels measured using ICP‐MS in the serum (B), duodenum (B’), and liver (B’’) of the indicated mice at dpi6. n = 6–9 mice per group. C) Summary of *Mt1* mRNA measured in IECs isolated from the indicated mice at dpi6. n = 4 mice per group. D) Representative fluorescence images of villi (top row) and crypts (bottom row) in small intestine sections prepared from *Slc30a1^Flag‐eGFP^* mice and immunostained with anti‐Flag antibody (red). Also shown is the EGFP channel (green), and the nuclei were counterstained with DAPI (blue). E) *Z*‐stacked fluorescence images of organoids prepared from *Slc30a1^Flag‐eGFP^* mice and immunostained with anti‐Flag antibody (red); the nuclei were counterstained with DAPI (blue). F) Frame displayed images of organoids prepared from *Slc30a1^Flag‐eGFP^* mice and immunostained with anti‐Flag (red) and anti‐Actin (green) antibodies; for each organoid, the lumen is indicated (“L”). G) Schematic diagram depicting the process by which Slc30a1 regulates systemic zinc via its localization to the basolateral membrane of IECs. H) Representative images of propidium iodide (PI)—stained cells in organoids derived from *Slc30a1^iIEC‐KO^* mice and treated for 48 h with EtOH or 4‐OHT, merged with the corresponding bright‐filed images. I) Summary of cell viability measured in organoids derived from *Slc30a1^iIEC‐KO^* mice and treated for 72 h with EtOH or 4‐OHT (n = 6 biological replicates per group). J) Schematic diagram depicting the strategy for treating mice with ZnSO_4_ after tamoxifen injections. K) Survival curve of the indicated mice; where indicated, the mice received zinc supplementation or vehicle. n = 6 mice per group. L–N) Summary of serum zinc levels (L), *Mt1* mRNA measured in IECs (M), and colon length (N) in the indicated mice at dpi6. n = 4–9 mice per group. O and P) Summary of serum ALT (O) and AST (P) levels measured at dpi6 in the indicated mice. n = 6 mice per group. Q) Representative images of small intestine sections obtained from the indicated mice at dpi6 and stained with H&E. R) Summary of *Lgr5* mRNA measured in IECs isolated from the indicated mice at dpi6. n = 7–10 mice per group S,T) Representative images of TUNEL‐stained small intestine sections obtained from the indicated mice at dpi6 (S) and summary of TUNEL‐positive cells per crypt (T) (n = 10 crypts pooled from 3 biological replicates per group). U) Summary of *Irf7* mRNA measured in IECs isolated from the indicated mice at dpi6. n = 4–7 mice per group V–X) Summary of the indicated cytokines measured in the serum of the indicated mice at dpi6. n = 5–6 mice per group. *p < 0.05, **p < 0.01, ***p < 0.001, and n.s., not significant; Student's *t‐*test (A–C and I), one‐way ANOVA with Tukey's post hoc test (L–P, R, and T–X), or log‐rank (Mantel‐Cox) test (K). Scale bars, 50 µm. See also Figures S8 and S9 (Supporting Information).

Slc30a1 is a known zinc transporter; therefore, to further examine how the loss of intestinal Slc30a1 causes an overwhelming inflammatory response and crypt cell death, we systemically studied the function of Slc30a1 in regulating zinc homeostasis. Using inductively coupled plasma mass spectrometry (ICP‐MS) to measure zinc levels revealed significantly lower levels of zinc in the serum of *Slc30a1^iIEC‐KO^* mice at both dpi3 and dpi6 compared to control mice (**Figure** **4**A,B), as well as increased levels of zinc in the duodenum (Figure 4A,B). Interestingly, however, zinc levels in the spleen were similar between *Slc30a1^iIEC‐KO^* and control mice (Figure S8A,B, Supporting Information), but hepatic zinc levels were significantly lower in the *Slc30a1^iIEC‐KO^* mice at dpi6 (Figure 4A,B). Consistent with these results, we also measured significantly higher levels of *Mt1* mRNA (encoding metallothionein 1, a surrogate marker of intracellular zinc levels)^[^ ^48^, ^49^ ^]^ in IECs in *Slc30a1^iIEC‐KO^* mice compared to control mice (Figure 4C).

Next, we examined the macroscopic and subcellular localization of Slc30a1 in IECs by generating a transgenic mouse line in which the endogenous *Slc30a1* gene is tagged with a 3 × Flag tag and releasable enhanced‐GFP (EGFP) in order to visualize Slc30a1 expression (*Slc30a1^Flag‐eGFP^*; Figure S8C, Supporting Information). We found that EGFP fluorescence was visible throughout the small intestine in *Slc30a1^Flag‐eGFP^* mice (Figure 4D), suggesting that *Slc30a1* is widely expressed in IECs. Moreover, Flag immunofluorescence confirmed the presence of Slc30a1 in the basolateral compartment of IECs (Figure 4D). To support this finding, we isolated crypt cells from *Slc30a1^Flag‐eGFP^* mice and seeded these cells within Matrigel matrix domes in order to grow 3D organoids (Figure S8D, Supporting Information). Using mature intestinal organoids derived from *Slc30a1^Flag‐eGFP^* mice, Flag immunofluorescence confirmed the presence of Slc30a1 in the basolateral compartment of IECs (Figure 4E,F; Figure S8D,E, Supporting Information). These results indicate that intestinal Slc30a1 localizes to the basolateral side of IECs, where it governs the uptake of intestinal zinc, and its loss results in zinc overload in the IECs together with zinc deficiency in the circulation (Figure 4G).

Notably, treating organoids derived from *Slc30a1^iIEC‐KO^* mice with 4‐hydroxytamoxifen (4‐OHT) to induce *Slc30a1* knockout triggered massive cell death compared to vehicle (EtOH)–treated organoids (Figure 4H,I; Figure S8D,F,G, Supporting Information), even in the presence of the zinc chelator TPEN (*N,N,N’,N’*‐Tetrakis‐(2‐pyridylmethyl)‐ethylenediamine) (Figure S8H, Supporting Information). In contrast, giving *Slc30a1^iIEC‐KO^* mice intraperitoneal (i.p.) injections of ZnSO_4_ (Figure 4J) prevented the effects of knocking out intestinal *Slc30a1*, and this protection lasted as long as the mice continued to receive ZnSO_4_ injections, as the mice died soon after the ZnSO_4_ injections stopped (Figure 4K; Figure S9A, Supporting Information). We also found that oral gavage of ZnSO_4_ had no effects on the survival of *Slc30a1^iIEC‐KO^* mice, which is consistent with our finding that intestinal Slc30a1 is unique for circulatory zinc complement. Although i.p. ZnSO_4_ injections increased serum zinc levels in *Slc30a1^iIEC‐KO^* mice (Figure 4L), there was no significant effect on *Mt1* expression (Figure 4M) but with further upregulated mRNA expression of *Mt2* (Figure S9B–D, Supporting Information) in the IECs. Meanwhile, we observed a slightly increased trend of Mt1 protein abundance in vehicle‐ or ZnSO_4_‐treated *Slc30a1^iIEC‐KO^* mice (Figure S9E, Supporting Information). Moreover, in addition to preventing mortality, ZnSO_4_ injections significantly reduced morbidity in *Slc30a1^iIEC‐KO^* mice (Figure 4N–Q; Figure S9F–H, Supporting Information). Importantly, ZnSO_4_ injections also increased expression of the stem cell marker *Lgr5* in *Slc30a1^iIEC‐KO^* mice (Figure 4R) and reduced the number of dying cells in the crypt region (Figure 4S,T), suggesting that systemic zinc supplementation can suppress intrinsic cell death triggered by the loss of intestinal Slc30a1. Notably, we also found that zinc supplementation significantly reduced the expression of genes related to the inflammatory response (Figure 4U; Figure S9I–K, Supporting Information), and suppressed the cGAS‐STING pathway (Figure S9L,M, Supporting Information) and serum cytokine levels (Figure 4V–X; Figure S9N, Supporting Information) in *Slc30a1^iIEC‐KO^* mice. Together, these results suggest that reduced systemic zinc levels contribute to the lethal phenotype observed in intestine‐specific *Slc30a1* knockout mice, indicating that intestinal Slc30a1 is essential for the maintenance of systemic zinc homeostasis.

### 2.5 Cryo‐EM Structure of Human SLC30A1

**Figure 5:**
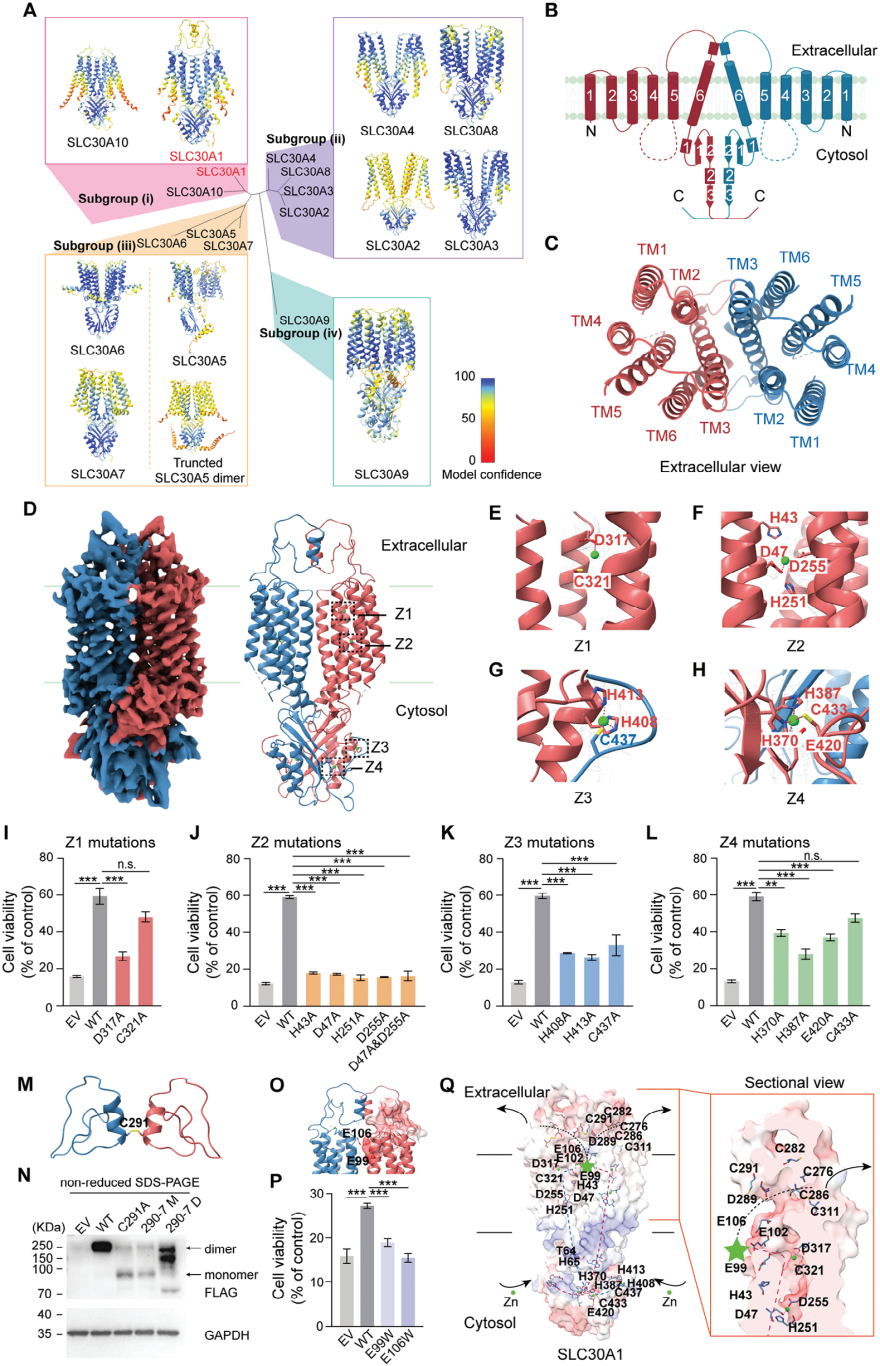
Cryo‐EM structure of human SLC30A1. A) Predicted structures of all ten human SLC30A proteins, with the model confidence indicated using a color scale. The ten structures are classified into four subgroups based on the phylogenetic tree. Except for SLC30A5, the full‐length proteins were used to predict the structure; the dimer structure of SLC30A5 in subgroup (iii) was predicted using a truncated version lacking the first 398 residues. Also shown is the predicted structure of the SLC30A5 monomer. Model confidence = 0.8*ippTM+0.2*pTM. B) 2D structure of the human SLC30A1 dimer. The two protomers are shown in red and blue. α helices are depicted as cylinders, and β sheets are depicted as arrows. C) Extracellular (top‐down) view of the TM domains in the SLC30A1 dimer. D) Density map (left) and tertiary structure (right) of human SLC30A1 based on cryo‐EM, showing the approximate locations of the four zinc‐binding sites (Z1 through Z4) indicated in one of the two protomers. The two protomers are shown in blue and red. E) The Z1 zinc‐binding site is located in TM6 and is comprised of residues D317 and C321. F) The Z2 zinc‐binding site connects TM2 and TM5 via the His43, Asp47, His251, and Asp255 residues. G) The Z3 zinc‐binding site connects the cytosolic domains of the two SLC30A1 protomers via the His408 and His413 residues. H) The Z4 zinc‐binding site is located in the cytosolic domain and is comprised of the His370, His387, Glu420, and Cys433 residues in each protomer. I–L) SLC30A1‐KO HeLa cells were transfected with an EV, wild‐type SLC30A1, or SLC30A1 with the indicated mutations in Z1 (I), Z2 (J), Z3 (K), and Z4 (L). The transfected cells were then exposed to high extracellular zinc, and cell viability was measured and is expressed relative to non‐zinc‐treated cells (n = 3 biological replicates per group). M) Detailed structure of the extracellular dimer interface in SLC30A1, showing the C291 residues in each protomer forming a disulfide bond. N) Western blot analysis of lysates prepared from cells expressing an EV, WT SLC30A1, the C291A mutant, a mutant in which residues 290–297 were replaced with Gly‐Gly‐Ala‐Gly‐Gly‐Ala‐Gly‐Gly residues (290‐7 M), and a mutant in which residues 290–297 were deleted (290‐7 D). The proteins were separated using non‐reducing SDS‐PAGE. O) Detailed view of residues E99 and E106 located near the end of the TM helices. Negative electrostatic potential was used to identify the pathway for zinc release from the extracellular domain. P) Summary of zinc toxicity measured in cells expressing WT SLC30A1 or the indicated SLC30A1 mutants (n = 3 biological replicates per group). Q) Proposed zinc transport pathway for human SLC30A1. Shown on the left is the electrostatic potential map, with red and blue representing negative and positive potential, respectively. The indicated residues refer to the zinc‐binding sites predicted using MIB, as well as the sites indicated by cryo‐EM. Shown on the right is a cross‐sectional detailed view of the proposed path by which the zinc ions exit the transporter; the green star indicates the site at which zinc ions converge upon exiting the TM bundles. **p < 0.01, ***p < 0.001, and n.s., not significant; one‐way ANOVA with Tukey's post hoc test. See also Figures S10–S15 (Supporting Information).

Given that SLC30A1 is highly conserved among vertebrates (Figure S10, Supporting Information), we examined the structure of human SLC30A1 and investigated the molecular mechanism underlying its zinc transport function. We first predicted the structures of all ten human SLC30A family members by analyzing AlphaFold2‐Multimer using the COSMIC platform;^[^ ^50^ ^]^ the predicted secondary structures of SLC30A1 through SLC30A10 are shown in Figure S11 (Supporting Information), revealing relatively conserved C‐terminal structures and non‐conserved transmembrane domains (TMDs). These structures were then classified into four subgroups according to a phylogenetic tree based on their protein sequences (**Figure** **5**A; Figure S12, Supporting Information). Although the predicted structures of SLC30A3, SLC30A4, SLC30A6, SLC39A8, and SLC30A9 had relatively high confidence, AlphaFold2‐Multimer failed to predict the structure for the other five SLC30A members, including SLC30A1 (Figure 5A). Therefore, we expressed and purified the full‐length human SLC30A1 protein and then resolved the structure at 3.7‐Å resolution using cryo‐EM (Figure S13 and Tables S1,S2, Supporting Information). The resulting structure indicates that SLC30A1 forms a homodimer, with each subunit consisting of a TMD with six transmembrane helices (TM1‐TM6), an extracellular domain (ECD), and an intracellular C‐terminal domain (CTD) (Figure 5B). The overall structure shows that the SLC30A1 homodimer is relatively compact, with the TM helices in each subunit forming symmetrical helical bundles and TM2, TM3, and TM6 contributing to the formation of the dimer interface (Figure 5B,C). Under basal conditions, we found that each protomer in the SLC30A1 dimer contains four putative zinc‐binding sites (Z1 through Z4) located in the TMD and CTD (Figure 5D–H). The Z1 and Z2 zinc‐binding sites are located in the TMD, possibly forming the zinc transport pathway; in contrast, Z3 and Z4 are located in the CTD, where they likely sense intracellular free zinc ions and form the starting point for zinc transport. Among the four zinc‐binding sites, Z2 is the most conserved, with a zinc ion connecting TM2 and TM5 via four coordinated residues (His43, Asp47, His251, and Asp255) (Figure 5F). Moreover, a zinc ion in Z3 stabilizes the CTD‐CTD interaction via His408 and His413 in one protomer and Cys437 in the other protomer (Figure 5G). Finally, the Z4 site resides in a highly negatively charged cavity comprised of His370, His387, Glu420, and Cys433 (Figure 5H).

We then confirmed the putative zinc‐binding sites identified in the cryo‐EM structure by measuring zinc transport in SLC30A1‐KO HeLa cells (in which endogenous *SLC30A1* expression is knocked out) transfected with an empty vector (EV), wild‐type SLC30A1, or SLC30A1 containing various mutations (Figure S14A,B, Supporting Information). By indirectly quantifying zinc transport under high zinc levels—in which increased cell viability reflects increased zinc efflux via SLC30A1^[^ ^32^ ^]^—we found that mutations in Z2 had the most robust effect on reducing the transport function of SLC30A1 (Figure 5I–L; Figure S14C, Supporting Information). Moreover, loading cells with the fluorescent zinc indicator FluoZin‐3 AM revealed increased intracellular zinc concentration in cells expressing SLC30A1 with mutations in Z2, consistent with reduced zinc export (Figure S14D, Supporting Information). We also found that Cys291 in the helical domain in the ECD of each protomer forms a disulfide bond, which stabilizes the dimer conformation and is essential for SLC30A1 function (Figure 5M,N; Figure S14E, Supporting Information).

Next, using the Metal Ion‐Binding Site Prediction and Docking Server (MIB),^[^ ^51^ ^]^ we identified additional putative zinc‐binding sites in SLC30A1. The electrostatic potential indicates a channel that mediates the release of zinc from the site of convergence into the extracellular space (Figure 5O). Moreover, introducing either the E99W or E106W mutation reduced the zinc transport function of SLC30A1 (Figure 5P), presumably by blocking the zinc transport pathway. We, therefore, propose a model for the zinc transport pathway for SLC30A1 based on electrostatic potential and zinc‐binding sites (Figure 5Q); this model of zinc efflux reflects a novel dimer‐dependent mode of zinc transport that may be unique to SLC30A1 and has not been reported previously for any other cation diffusion facilitators.

### 2.6 The H43 Residue in SLC30A1 Mediates Zinc Selectivity

Among the SLC30A family of transporters, SLC30A1 is most closely related to SLC30A10 (Figure 5A; Figure S15A, Supporting Information); however, SLC30A10 has been reported to transport manganese.^[^ ^52^, ^53^ ^]^ To investigate the mechanism underlying the putative difference in ion selectivity between these two transporters, we predicted the structure of the SLC30A10 monomer based on the SLC30A1 structure using the homology‐modeling server SWISS‐MODEL^[^ ^54^ ^]^ (Figure S15B, Supporting Information). We found that nearly all of the amino acid residues in the zinc‐binding sites in SLC30A1 are conserved in SLC30A10, with the exceptions of His43 and Cys321 (Figure S15A, Supporting Information). Given that Cys321 in SLC30A1 has a similar chemical property and orientation as its corresponding residue (Thr284) in SLC30A10 (Figure S15C, Supporting Information), we hypothesized that His43 in SLC30A1 (corresponding to Asn43 in SLC30A10) likely plays a predominant role in the transporter's ion selectivity. We found that SLC30A1‐KO HeLa cells expressing SLC30A1 with the H43N mutation (Figure S15D, Supporting Information) had increased sensitivity to zinc toxicity and reduced sensitivity to manganese toxicity compared to cells expressing WT SLC30A1 (Figure S15E, Supporting Information); conversely, cells expressing SLC30A10 with the N43H mutation (Figure S15D, Supporting Information) had reduced sensitivity to zinc toxicity and increased sensitivity to manganese toxicity (Figure S15E, Supporting Information). Moreover, when we loaded the cells with FluoZin‐3 AM to measure intracellular zinc concentration upon application of exogenous zinc, we found that transporters containing a His residue at position 43 preferentially transported zinc out of cells to reduce intracellular zinc (Figure S15F,G, Supporting Information). These results suggest that the identity of the amino acid at position 43 plays a key role in determining the transporter's selectivity for zinc versus manganese, possibly due to differences in steric hindrance based on the functional group.

## 3 Discussion

Here, we functionally characterized the essential role of intestinal SLC30A1 in regulating systemic zinc homeostasis. Interestingly, we found that SLC30A1 is localized to the basolateral membrane of the intestine, where it controls zinc efflux from enterocytes into the circulation. Mechanistically, we found that intestinal SLC30A1 regulates gut barrier integrity and systemic zinc homeostasis. Most importantly, we also provide the structure of human SLC30A1, which not only revealed a unique dimer‐dependent zinc transport mode, but also identified the His43 residue as the principal residue regulating ion selectivity in SLC30A1.

The distribution of zinc ions in the body is tightly regulated by a precise, highly coordinated network of metal transporters (SLC30A and SLC39A family members) and storage proteins such as metallothioneins. Here, we show that SLC30A1 in the basolateral membrane of IECs plays a central role in this network. Based on our results, SLC30A1 transports zinc primarily via cells in the villi, which express higher levels of SLC30A1 compared to cells in the crypt. Interestingly, the death of IECs was restricted to the crypt region in our animal models, suggesting that crypt‐based cells are more sensitive to the loss of SLC30A1. While villus cells intrinsically undergo senescence and anoikis (a form of programed cell death that occurs upon detachment from the extracellular matrix) in a few days, the crypt consists of undifferentiated cells and serves as the source of new IECs.^[^ ^55^ ^]^ Cell proliferation and cell differentiation require large amounts of newly biosynthesized proteins and nucleic acids, as well as zinc ions, and insufficient zinc has been shown to trigger cell cycle arrest.^[^ ^56^, ^57^ ^]^ Thus, IECs in the crypt that undergo proliferation may be more susceptibility to low extracellular zinc; consistent with this hypothesis, we found that high zinc levels in IECs were not the cause of cell death while application of extracellular zinc significantly blocked the death of IECs.

Systemic zinc deficiency has been reported in patients with inflammatory bowel disease (IBD),^[^ ^58^, ^59^ ^]^ however, the causal relationship—if any—between low systemic zinc and IBD remains unclear, and key questions remain. E.g., do decreased zinc levels trigger IBD, or does the inflamed intestine cause insufficient zinc absorption? Interestingly, low serum zinc levels have been correlated with poor prognosis,^[^ ^60^, ^61^ ^]^ and zinc supplementation can improve clinical outcome^[^ ^60^, ^62^ ^]^ and reduce gut permeability in patients with CD, thereby protecting against relapse.^[^ ^63^ ^]^ Our results suggest that maintaining circulating zinc levels via the activity of intestinal SLC30A1 plays an essential role in maintaining intestinal integrity; moreover, serum zinc levels play a key role in determining the fate of IECs. However, loss of function of SLC30A1 is overwhelming, as its absence leads to embryonic death in mouse models. It is therefore reasonable to hypothesize that the recently identified intergenic mutation in the *RD3‐SLC30A1* locus in male patients with CD might affect SLC30A1‐mediated zinc absorption, rather than abolishing the function of SLC30A1. Further studies are needed to test this hypothesis.

Although SLC30A1 was previously reported to be the sole zinc exporter ubiquitously expressed at the plasma membrane,^[^ ^64^ ^]^ a 35‐kDa isoform of SLC30A2 in mammary glands was later reported to serve as a transporter in the plasma membrane.^[^ ^65^ ^]^ A variant of SLC30A5 has also been shown to be expressed at the plasma membrane.^[^ ^66^ ^]^ Notably, in our study we found that no other SLC30A family members compensated for the loss of intestinal SLC30A1 in terms of transporting zinc ions into the systemic circulation, suggesting that intestinal SLC30A1 is solely responsible for this function. This unique transport function of SLC30A1 is reminiscent of ferroportin (FPN), the sole iron exporter, which has an essential role in mediating iron absorption in the intestine.^[^ ^67^ ^]^

By analyzing the molecular structures of SLC30A1 and its closest SLC30A family member—the manganese transporter SLC30A10—we found that His43 in SLC30A1 plays a critical role in determining the transporter's selectivity for zinc ions. However, a recent study found that somatic mutations in SLC30A1 close to His43 are associated with aldosterone‐producing adenomas and primary aldosteronism by altering intracellular calcium levels, further increasing the production of aldosterone.^[^ ^34^ ^]^ Future studies are warranted in order to better understand the precise transport mechanisms involved in various pathological conditions. Nevertheless, our study provides valuable new insights into the transport function of SLC30A1 and may facilitate the development of new therapeutic strategies that target SLC30A1.

## 4 Conclusion

In conclusion, our results uncovered the tissue‐specific characteristic of Slc30a1, clearly elucidated the essential role of intestinal Slc30a1 in zinc homeostasis, and displayed the physiological location of the protein that is important for its function. We believe that further studies based on our findings and establishment of human SLC30A1 structure would help understand the pathological relevance of SLC30A1 with diseases, such as cancers, as well as propose new insights in regulating systemic zinc homeostasis by manipulating SLC30A1 for therapeutic purpose.

## 5 Experimental Section

### 5.1 Animal Experiments

#### 5.1.1 Animals

All animal experiments were approved by the Institutional Animal Care and Use Committee of Zhejiang University and were performed in accordance with the committee's guidelines (No. ZJU20240655).

*Slc30a1* floxed mice were generated by Shanghai Biomodel Organisms Center, Inc. (Shanghai, China). The gene‐targeting strategy used to conditionally excise exon 2 in the mouse *Slc30a1* gene is shown in Figure S1A (Supporting Information). Heterozygous *Slc30a1* floxed (*Slc30a1^flox/+^*) mice were backcrossed to the C57BL/6 background for more than five generations. To generate global inducible *Slc30a1* knockout mice (*Slc30a1^iKO^*), *Slc30a1^flox/flox^* mice were crossed with *UBC‐Cre^ERT^* transgenic mice. To generate intestine‐specific *Slc30a1* knockout mice (*Slc30a1^IEC‐KO^*), *Slc30a1^flox/flox^* mice were crossed with *Villin‐Cre* transgenic mice (Figure S2A, Supporting Information); the *Villin‐Cre* transgenic mice were kindly provided by Dr. Chaofeng Wang (Army Medical University, Chongqing, China). To generate inducible intestine‐specific *Slc30a1* knockout mice (*Slc30a1^iIEC‐KO^*), *Slc30a1^flox/flox^* mice were crossed with *Villin‐Cre^ERT2^* transgenic mice (Shanghai Biomodel Organisms Center, Inc.) (Figure S2A, Supporting Information). *Slc30a1^Flag‐eGFP^* mice were generated by Shanghai Biomodel Organisms Center, Inc (Figure S8C, Supporting Information). For experiments involving knockout mice, *Slc30a1^flox/flox^* littermates were used as controls. *Ripk3^−/−^* mice were originally generated by Dr. Xiaodong Wang (National Institute of Biological Sciences, Beijing), and were provided by Dr. Yan Zhang and Dr. Rui‐Ping Xiao (Peking University). *Mlkl^−/−^* and *Mlkl^−/−^;Fadd^−/−^* mice were provided by Dr. Haibing Zhang (Shanghai Institutes for Biological Sciences). Conditional *Slc7a11^Cas9‐TG^* transgenic mice were generated by GemPharmatech (Nanjing, China).

Unless indicated otherwise, the mice were fed a standard laboratory chow diet (Research Diets, New Brunswick, NJ) with free access to food and drinking water. All animals were housed at 23 ± 1 °C under a 12:12 h light/dark cycle. Genomic DNA was extracted from mouse tail biopsies using the Easy Tissue and Blood DNA Extraction Kits (#DR0301250, Easy‐Do, Hangzhou, China) and used for genotyping with the primer pairs listed in Table S3 (Supporting Information).

For tissue sample collection, mice were sacrificed under pentobarbital anesthesia at day 3, 6, or 10 post tamoxifen induction (dpi3, dpi6, or dpi10, respectively); blood was extracted by cardiac puncture.

#### 5.1.2 Drug Administration

For targeted gene deletion in the inducible knockout mice (*Slc30a1^iKO^* and *Slc30a1^iIEC‐KO^*), at 8–10 weeks of age the mice received five daily i.p. injections of tamoxifen (100 mg k^−1^g body weight; #T832955, Macklin Biochemical, Shanghai, China). Prior to injection, 10% (w/v) tamoxifen was dissolved in 99.9% EtOH, then brought to a final concentration of 10 mg mL⁻^1^ with corn oil. Where indicated, mice received daily i.p. injections of GSK’872 (20 mg k^−1^g body weight; #8465, Selleck Chemicals), disulfiram (25 mg k^−1^g body weight; #S1680, Selleck Chemicals), Fer‐1 (1 mg k^−1^g body weight; #S7243, Selleck Chemicals), or H‐151 (10 mg k^−1^g body weight; #S6652, Selleck Chemicals), or the corresponding vehicles; these injections began one day before the start of tamoxifen injections and continued throughout the experiment. The interval between injections of the various drugs and tamoxifen was at least at 4 h. Where indicated, mice received daily oral doses of emricasan (1 mg k^−1^g body weight; #S775, Selleck Chemicals) or vehicle starting 24 h before the first injection of tamoxifen and then continuing throughout the experiment. Where indicated, mice received an i.p. injection of 1.6 mM ZnSO_4_ (#Z0251, Sigma‐Aldrich) (10 µL g⁻^1^ body weight), which is determined based on our preliminary experiments, or vehicle (saline) daily throughout the experiment. Where indicated, the antibiotic cocktail (ABX) consisting of 1 g L⁻^1^ neomycin sulfate (#MB1716, MeilunBio, Dalian, China), 1 g L⁻^1^ ampicillin sodium (#MB1378, MeilunBio), 1 g L⁻^1^ metronidazole (#MB2200, MeilunBio), and 500 mg L⁻^1^ vancomycin (#V8050, Solarbio, Beijing, China), was added to the drinking water starting one week before tamoxifen injection and then continuing throughout the experiment.

#### 5.1.3 Blood and Serum Assays

Blood samples were collected in heparin‐pretreated microtubes, and hematological parameters were measured using an XN‐1000 V Hematology Analyzer (Sysmex, Japan) at the Center for Drug Safety Evaluation and Research, Zhejiang University. For serum, blood samples were collected and centrifuged for 15 min at 3000 rpm, and serum ALT, AST, LDH, and electrolyte levels were measured using a COBASC 311 Chemistry analyzer (Roche, Switzerland) at the Center for Drug Safety Evaluation and Research, Zhejiang University.

#### 5.1.4 Histology

Tissue samples were fixed overnight in 4% formaldehyde, embedded in paraffin, and serially sectioned at 5‐µm thickness. Where indicated, sections were stained with H&E for routine histological examination, periodic acid‐Schiff (PAS) to visualize goblet cells, or Masson's trichrome to detect extracellular matrix fiber. TUNEL staining to detect cell death was performed using the In Situ Cell Death Detection Kit (#1164817910, Roche) in accordance with the manufacturer's instructions. Bright‐filed images of stained sections were obtained using a Nikon ECLIPSE Ni‐U microscope.

#### 5.1.5 Transmission Electron Microscopy

Samples of small intestine (≈0.5 cm in length) were quickly removed and immediately fixed in 3% phosphate‐glutaraldehyde. The fixed samples were then post‐fixed, embedded, sectioned, and mounted at the Electron Microscopy Core Facility of Zhejiang University. The samples were visualized using a Tecnai 10 transmission electron microscope (Philips).

#### 5.1.6 In Vivo BrdU Labeling

To detect proliferating intestinal cells, in vivo bromodeoxyuridine (BrdU) labeling was performed using a BrdU Flow Kit (#559616, BD Biosciences) in accordance with the manufacturer's instructions. In brief, the mice received a single i.p. injection of BrdU (100 mg k^−1^g body weight) and were sacrificed 2, 24, or 48 h after injection. Intestine samples were harvested, fixed, sectioned, stained with anti‐BrdU antibody, and imaged using a Nikon ECLIPSE Ni‐U microscope.

#### 5.1.7 Immunohistochemistry

Sections of intestine sample were deparaffinized and blocked with PBS containing 5% (v/v) normal goal serum and 1% (w/v) BSA. The sections were then incubated with primary antibodies overnight at 4 °C, followed by incubation with the species‐appropriate secondary antibodies for 1 h at room temperature. The following primary antibodies were used: anti‐Slc30a1 (custom‐produced by ABclonal Technology, Wuhan, China), anti‐Ki67 (#ab15580, Abcam), anti‐Lyz1 (#60487, Cell Signaling Technology), and anti‐Cleaved Caspase 3 (#9661, Cell Signaling Technology). Bright‐field images were obtained using a Nikon ECLIPSE Ni‐U microscope.

For immunofluorescence, frozen small intestine samples obtained from *Slc30a1^Flag‐eGFP^* mice were embedded in OCT agent, and sections were cut on a cryomicrotome. Anti‐Flag antibody (#A8592, Sigma‐Aldrich) was used to detect Flag‐labeled Slc30a1, and images were captured using a Zeiss LSM 900 confocal microscope and analyzed using ImageJ (NIH).

#### 5.1.8 Isolation of IECs

Immediately after the mice were sacrificed, intestine samples (≈5–10 cm in length) were harvested, opened longitudinally, washed with cold Hanks balanced salt solution (HBSS), cut into 5‐mm pieces, and incubated in HBSS containing 30 mM EDTA for 20 min at 4 °C. After incubation, the samples were washed three times with HBSS and shaken to release the epithelial cells into suspension. For subsequent RNA or protein extraction, the suspended epithelial cells were collected by centrifugation at 800 rpm for 10 min at 4 °C. Where indicated, crypt and villus cells were separated for analysis. Specifically, the cell suspension was passed through a 70‐µm cell strainer, and crypt cells were collected by centrifugation at 800 rpm for 10 min at 4 °C. After discarding the lamina propria and muscularis mucosa, the villus cells remaining on the cell strainer were flushed into a new centrifuge tube using HBSS and collected by centrifugation at 800 rpm for 10 min at 4 °C.

#### 5.1.9 Real‐Time PCR Analysis

Total RNA was extracted using TRIzol reagent (#ET111‐01‐V2, TransGen Biotech, Beijing, China). The concentration and purity of the total RNA was measured using a NanoDrop 2000 Spectrophotometer (Thermo Fisher Scientific), and 1–2 µg RNA per sample was reverse‐transcribed to cDNA using the HiScript II 1^st^ Strand cDNA Synthesis Kit (#11123ES, Yeasen Biotech, Shanghai, China). Real‐time PCR reactions were performed in duplicate for each sample using Hieff UNICON Universal Blue qPCR SYBR Green Master Mix (#1184ES, Yeasen Biotech, Shanghai, China) in a LightCycler 480 Real‐Time PCR System (Roche) using the primers listed in Table S3 (Supporting Information).

#### 5.1.10 RNA Sequencing and Data Analysis

Whole‐genome gene expression analysis was performed on IECs. Total RNA was extracted as described above, and ≈1–3 µg RNA per sample was used for analysis. Sequencing libraries were generated using VAHTS Universal V6 RNA‐seq LibraryPrep Kit for Illumina (#NR604‐01/02, Vazyme) in accordance with the manufacturer's instructions, and index codes were added to attribute sequences to each sample. Clustering of the index‐coded samples was performed on a cBot cluster generation system using HiSeq PE Cluster Kit v4‐cBot‐HS (Illumina) in accordance with the manufacturer's instructions. After cluster generation, the libraries were sequenced on an Illumina platform, and 150‐bp paired‐end reads were generated. Both cluster generation and sequencing were performed on a NovaSeq 6000 S4 platform, using Reagent kit V1.5. Raw reads were filtered to produce the clean data. Differentially expressed genes were identified based on a |log_2_(fold change)| ≥1 and an adjusted *p*‐value of <0.05. All differentially expressed genes were used for heat map analysis and KEGG ontology enrichment analysis. For analysis of mRNA in crypts obtained from mice at dpi6, one biological replicate was excluded due to clustering anomaly.

#### 5.1.11 Western Blot Analysis

Total proteins were extracted using RIPA buffer (#R0020, Solarbio) containing protease inhibitor cocktail (EDTA‐free) (#HY‐K0010, MCE) and phosphatase inhibitor (#04906845001, Roche). The sample homogenate was centrifuged at 12000 rpm, and the supernatant was collected for protein analysis. Protein concentration was measured using an Enhanced BCA Protein Assay Kit (#P0010, Beyotime Biotechnology, Shanghai, China), and a total 30 or 50 µg protein per sample was separated in a 10% SDS‐PAGE gel and then transferred to a PVDF membrane (#1620174, Bio‐Rad). The membranes were blocked with 5% (w/v) BSA or skim milk in TBST (Tris‐buffered saline containing 0.1% Tween‐20) for 2 h at room temperature, washed three times for 10 min each with TBST, and then incubated with primary antibodies under gentle agitation overnight at 4 °C. The following primary antibodies were used: anti‐Slc30a1 (1:1500, custom‐produced by ABclonal Technology, Wuhan, China), anti‐Mlkl (1:1000, #37705, Cell Signaling), anti‐phospho‐Mlkl (1:1000, #7420, Affinity Biosciences), anti‐Nrf2 (1:1000, ab137550, Abcam), anti‐Heme oxygenase 1 (1:1000, ab13243, Abcam), anti‐Gpx4 (1:1000, #67763‐1‐lg, Proteintech), anti‐Acsl4 (1:500, sc‐365230, Santa Cruz), anti‐Irf3 (1:1000, ab68481, Abcam), anti‐phospho‐Irf3 (1:1000, #4947, Cell Signaling), anti‐STING (1:1000, ab288157, Abcam), anti‐Stat1 (1:1000, #14994, Cell Signaling), anti‐phospho‐Stat1 (1:1000, #7649, Cell Signaling), anti‐Stat3 (1:1000, #9139, Cell Signaling), anti‐phospho‐Stat3 (1:1000, #9138, Cell Signaling), anti‐Jak2 (1:1000, #3230, Cell Signaling), anti‐phospho‐Jak2 (1:1000, #3771, Cell Signaling), anti‐p65 (1:1000, ab16502, Abcam), anti‐phospho‐p65 (1:1000, #3033, Cell Signaling), anti‐MT1X (1:1000, #17172‐1‐AP, Proteintech), and anti‐β‐Actin (1:50000, AC026, ABclonal Technology). The membranes were then washed three times for 10 min each with TBST, and then incubated with the species‐appropriate HRP‐conjugated secondary antibodies (1:3000, #AS003 and #AS014, ABclonal Technology) for 90 min at room temperature. After three washes for 10 min each with TBST, the signals were detected using either the Pierce ECL Western Blotting Substrate (#32106, Thermo Scientific) or the SuperSignal West Pico PLUS system (#34580, Thermo Scientific), with the Bio‐Rad ChemiDoc Touch Imaging System. The signals were analyzed using Image Lab 6.1 software (Bio‐Rad Laboratories).

#### 5.1.12 FD‐4 Test

To measure intestinal permeability, the 4‐kDa fluorescent dextran‐FITC (FD‐4) test was used. Mice were fasted for 12 h and then given an oral dose (500 mg k^−1^g body weight) of FD‐4 (#FD4‐1G, Sigma‐Aldrich) in saline (50 mg mL⁻^1^). After an additional hour of fasting, the mice were sacrificed and blood samples were collected in heparinized microtubes via cardiac puncture. Plasma was obtained by centrifuging the blood samples at 12000 × *g* for 10 min at 4 °C and protected from light. A standard curve was obtained by serially diluting FD‐4 in plasma obtained from untreated mice. To measure FITC fluorescence, 100 µL of each sample and standard were transferred to a black 96‐well microplate (#3603, Corning), and fluorescence was measured using a SpectraMax M5 reader (Molecular Devices) with an excitation wavelength of 485 nm and an emission wavelength of 535 nm. FD‐4 concentration in the plasma samples was then determined using the standard curve.

#### 5.1.13 ICP‐MS

Trace elements were measured in serum and tissue samples using ICP‐MS. In brief, the samples were digested with EMSURE 65% nitric acid solution (#1.00456.2508, Merck) and diluted to a final volume of 2 mL or 5 mL with deionized water. The final diluted samples were analyzed using an Agilent 7800x ICP‐MS at the Analysis Center of Agrobiology and Environmental Sciences of Zhejiang University.

#### 5.1.14 MDA Measurement

Intestinal malondialdehyde levels were measured using a Lipid Peroxidation MDA Assay Kit (#S0131, Beyotime Biotechnology) in accordance with the manufacturer's instructions.

#### 5.1.15 ELISA

Serum levels of the cytokines TNFα, IFNα, IFNβ, and IFNγ were measured using the Mouse TNF‐α ELISA Kit (#E‐EL‐M3063, Elabscience), Mouse IFN‐alpha ELISA Kit (#RK06086, ABclonal Technology), Mouse IFN‐beta ELISA Kit (#00420, ABclonal Technology), and Mouse IFN‐gamma ELISA Kit (#ELM‐IFNg‐1, RayBiotech), respectively, in accordance with the manufacturers’ instructions.

#### 5.1.16 Isolation of Crypts and Culture of Intestinal Organoids

Crypts were isolated from the small intestine and used for organoid cultures. In brief, a 20‐cm segment of small intestine proximal to the stomach was isolated from a sacrificed 6‐week‐old mouse. After the external membrane, vessels, and fat were removed, the intestinal segment was opened longitudinally and gently washed with cold PBS. The clean intestine segment was then cut into 2‐mm pieces and transferred to a 50‐mL conical tube containing 15 mL cold PBS. After washing 15–20 times with cold PBS, the intestinal pieces were suspended in 25 mL room temperature Gentle Cell Dissociation Reagent (#100‐0485, STEMCELL Technologies) and incubated at room temperature for 15 min with gentle rotation. After incubation, the intestine pieces were allowed to settle by gravity, the supernatant was discarded, 10 mL cold PBS containing 0.1% (w/v) BSA was added to the tube, and the intestine pieces were triturated through a pipette to release the crypts. The suspension was then filtered through a 70‐µm cell strainer, and the crypts were collected by centrifugation at 300 × *g* for 5 min at 4 °C. The crypt‐containing pellets were washed once in cold PBS containing 0.1% BSA and then resuspended in 10 mL cold DMEM/F‐12 (#L310KJ, BasalMedia, Shanghai, China). After counting the number of crypts in a 10‐µL aliquot, an appropriate volume containing ≈1500 crypts (enough crypts to seed four wells of a 24‐well cell culture plate) was aliquoted and centrifugated at 300 × *g* for 5 min at 4 °C. The collected crypts were resuspended in 150 µL room temperature complete IntestiCult Organoid Growth Medium (#06005, STEMCELL Technologies) containing penicillin/streptomycin (100 U/100 µg per mL) and 150 µL undiluted Matrigel Matrix (#356231, Corning) on ice. The mixture containing the crypts was then seeded on four wells in a 24‐well cell culture plate, and the plate was carefully placed in a 37 °C incubator for 10 min to set the Matrigel domes. Finally, 500 µL of room temperature complete IntestiCult Organoid Growth Medium containing penicillin/streptomycin (100 U/100 µg per mL) was added to each well. During culture, the medium was exchanged completely every four days, and the organoids were passaged every 7–10 days with a 1:6 split ratio.

For subsequent experiments, organoids derived from *Slc30a1^iIEC‐KO^* mice were allowed to grow for 48 h and were then treated with either 1 µM 4‐hydroxytamoxifen (4‐OHT) (#S7827, Selleck Chemicals) to induce gene knockout or ethanol (EtOH) for another 48 h. Total RNA was then extracted from the organoids in two combined wells of a 24‐well plate for each sample.

#### 5.1.17 Organoid Immunofluorescence

For organoid immunofluorescence, organoids were prepared from the small intestine of *Slc30a1^Flag‐eGFP^* mice as previously described,^[^ ^68^ ^]^ with modification. In brief, organoids seeded in 24‐well plates were fixed with 4% formaldehyde in cold PBS for 3 h at room temperature. After washing with cold PBS, the organoids were permeabilized with 0.5% Triton X‐100 in PBS for 1 h, and then blocked with 5% (v/v) normal goat serum in PBS containing 0.1% Triton X‐100 for 3 h. The organoids were then incubated with anti‐Flag antibody (1:200 diluted in blocking buffer) (#A8592, Sigma‐Aldrich) overnight at 4 °C, washed three times with PBS, and then incubated with Alexa Fluor 647‐labeled Goat Anti‐Mouse IgG (H+L) (1:500 diluted in blocking buffer; #A0473, Beyotime Biotechnology) for 2 h at room temperature. The cell nuclei were stained with 0.2 µg mL⁻^1^ DAPI (#C1005, Beyotime Biotechnology) in PBS for 2 h at room temperature. The organoids were then incubated with ActinRed 555 ReadyProbes Reagent (#R37112, Invitrogen) overnight at 4 °C, and images were captured using a Leica Stellaris 8 Confocal Microscope Platform and analyzed using ImageJ.

#### 5.1.18 Organoid Cell Viability Assay

For drug treatment, passaged organoids were seeded in a 2:3 mixture of Matrigel Matrix and complete IntestiCult Organoid Growth Medium in a 96‐well plate. The organoids were then cultured in 100 µL IntestiCult Organoid Growth Medium per well for 48 or 72 h, followed by an additional 48 h with 1 µM 4‐OHT (or EtOH) and *N,N,N’,N’*‐Tetrakis‐(2‐pyridylmethyl)‐ethylenediamine (TPEN) (#P4413, Sigma‐Aldrich) at the indicated concentrations. The culture medium was refreshed every 24 h. After treatment, 100 µL of room temperature CellTiter‐Glo 3D Reagent (#9681, Promega) was added to each well, and the Matrigel domes were disrupted by mechanical disruption. The 96‐well plates were then vigorously shaken on a plate shaker for 5 min, and then incubated at room temperature for 25 min. The contents were then transferred to a black 96‐well microplate with a transparent bottom (#3603, Corning), and luminescence was measured using a SpectraMax iD5 reader (Molecular Devices)

#### 5.1.19 PI Staining of Organoids

Organoids derived from *Slc30a1^iIEC‐KO^* mice were seeded and grown for 48 h, then cultured for an additional 48 h in 1 µM 4‐OHT (or EtOH). After removing the 4‐OHT/EtOH, the organoids were stained with 1 µg mL⁻^1^ propidium iodide (PI) (#MA0137, MeilunBio), and images were captured using a Nikon A1R confocal microscopy.

### 5.2 Structure Analysis

#### 5.2.1 Cell Culture

HEK293F cells were cultured in SMM 293‐T1 medium (#M293T1, Sino Biological Inc.) supplemented with penicillin‐streptomycin in a 37 °C orbital shaker rotating at 140 rpm in 8% CO_2_. HEK293T and HeLa cells were cultured in high‐glucose DMEM supplemented with 10% (v/v) FBS (Gibco) and penicillin‐streptomycin at 37 °C in 5% CO_2_.

#### 5.2.2 Protein Expression and Purification

The full‐length human SLC30A1 cDNA with codon optimization was cloned into the pEZT vector, and Strep‐tag II was added to the C‐terminal end of SLC30A1. The plasmid was then transfected into HEK293F cells using PEI; 18 h after transfection, 10 mM sodium butyrate was added to the culture medium to induce plasmid expression. 48 h after induction, the cells were collected by centrifugation at 1200 rpm and washed once with PBS. The cell pellet was snap‐frozen in liquid nitrogen and stored at ‐80 °C. To prepare cell lysates, the cell pellet was resuspended in lysis buffer containing 25 mM Tris (pH 8.0), 150 mM NaCl, 10% glycerol, 0.5% lauryl maltose neopentyl glycol (LMNG; #NG310, Anatrace), 0.1% cholesteryl hemisuccinate (CHS; #CH210, Anatrace), 1 × protease inhibitor cocktail, and 10 mg L⁻^1^ DNase I, and solubilized by rotating at 15 rpm at 4 °C for 3 h. The lysates were centrifugated at 40000 × *g* at 4 °C, and the supernatant was collected and incubated with Strep‐Tactin beads (IBA Lifesciences) rotating at 10 rpm at 4 °C for 2 h. The beads were then washed with wash buffer containing 25 mM Tris (pH 8.0), 150 mM NaCl, 10% glycerol, 0.01% LMNG, and 0.002% CHS to remove any nonspecific proteins from the beads. SLC30A1 was then eluted from the beads using elution buffer containing 25 mM Tris (pH 8.0), 150 mM NaCl, 10% glycerol 0.01% LMNG, 0.002% CHS, and 2.5 mM desthiobiotin (#D1411, Sigma‐Aldrich). The proteins were concentrated using ultrafiltration and further purified by gel filtration chromatography using a Superose 6 10/300 GL column (Cytiva) with SEC buffer containing 25 mM Tris (pH 8.0), 150 mM NaCl, 0.002% LMNG, and 0.0004% CHS. The appropriate fractions were collected, concentrated to 5 mg mL⁻^1^, and frozen. Vectors expressing mutant versions of SLC30A1 were generated using the Mut Express II Fast Mutagenesis Kit V2 (#C214, Vazyme).

#### 5.2.3 Cryo‐EM Grid Preparation and Data Collection

To prepare the cryo‐EM grids, 2.5 µL of purified wild‐type SLC30A1 (at 5 mg mL⁻^1^) was applied to glow‐discharged holey carbon grids (R1.2/1.3, 300 mesh, Quantifoil). The grids were then blotted for 3.0 s with a blot force of 5 at 4 °C in 100% humidity, and then plunge‐frozen in liquid ethane using a Vitrobot Mark IV (Thermo Fisher Scientific). Cryo‐EM imaging was then performed on a Titan Krios cryo‐EM microscope with 300 kV accelerating voltage at the Center for Cryo‐Electron Microscopy (Zhejiang University). Micrographs were recorded using a Gatan K2 Summit Detector in counting mode with a pixel size of 1.014 Å using SerialEM software.^[^ ^69^ ^]^ Image stacks were obtained at a dose rate of 8.0 electrons per Å^2^ per second with a defocus ranging from ‐1.0 to ‐2.5 µm. The total exposure time was 8 s, and 40 frames were recorded per micrograph. A total of 2713 movies were collected for WT SLC30A1.

#### 5.2.4 Image Processing and Map Construction

Dose‐fractionated image stacks were subjected to beam‐induced motion correction using MotionCor2.^[^ ^70^ ^]^ Contrast transfer function (CTF) parameters were estimated using Gctf v1.18.^[^ ^71^ ^]^ Particle selection and 2D and 3D classifications were performed on a binned dataset with a pixel size of 2.028 Å using RELION v.3.1.^[^ ^72^ ^]^

For WT SLC30A1, automated particle picking using Gaussian blob detection produced a total of 2 188 560 particles. These particles were then subjected to reference‐free 2D classification to discard fuzzy subsets of particles, producing 2 171 333 particles. A subsequent round of 3D classification focusing the alignment on the homodimer without detergent micelles produced one high‐quality subset containing 349 716 particles, which were re‐extracted into the original pixel size of 1.014 Å. 3D refinement with C2 symmetry imposed resulted in 3D reconstructions at 4.2‐Å resolution. To further improve the map quality, another round of 3D classification with alignment focusing on the CTD produced 204 108 particles, which were subsequently subjected to 3D refinement followed by local refinements of the ECD, TMD, and CTD domains at 3.2‐Å, 3.7‐Å, and 3.6‐Å resolution, respectively.

#### 5.2.5 Model Building and Refinement

The cryo‐EM structure of WT SLC30A8 (HsZnT8‐WT; PDB: 6XPE)^[^ ^73^ ^]^ was used as a starting model for rebuilding and refining the WT SLC30A1 structure against the EM density maps. The initial model was docked onto the cryo‐EM density map using the UCSF Chimera program.^[^ ^74^ ^]^ After the initial docked model was subjected to flexible fitting using Rosetta 2019.35,^[^ ^75^ ^]^ the models were subjected to iterative rounds of manual adjustment and auto refinement using Coot v.0.9.4^[^ ^75^ ^]^ and Phenix v.1.16,^[^ ^76^ ^]^ respectively. The final refinement scores were validated using the “comprehensive validation (cryo‐EM)” module in Phenix. The structures shown in the figures were generated using Chimera v.1.15^[^ ^74^ ^]^ and ChimeraX v.1.4.^[^ ^77^ ^]^

#### 5.2.6 Prediction of SLC30A Family Structures and Putative Zinc‐Binding Sites

AlphaFold2‐Multimer (version 2.2.0) was run in the COSMIC2 platform to predict the homodimer structure of SLC30A proteins by using two identical FASTA sequences as input files for each prediction,^[^ ^78^, ^79^, ^80^ ^]^ with the following databases: BFD (unmodified), MGnify: v2018_12 (unmodified), UniRef90: v2020_01, Uniclust30: v2018_08, UniProt: v2020_05. All sequences folded simultaneously in AlphaFold Multimer under the default settings. All predicted PDB models used Amber for relaxing in the last step in AlphaFold2‐Multimer. The output models were ranked using pLDDT, and the first model that provided the highest score was selected as the predicted model. Due to the complex structure of the SLC30A5 monomer, we used a slightly different procedure to predict the structure of the SLC30A5 homodimer. We first generated truncated versions of SLC30A5 lacking either residues 1–398 or residues 399–765; we then made predictions for homodimers based on both truncated proteins. The resulting dimer structure obtained for the dimer composed of the truncated protein lacking residues 1–398 was more confident than when we used a homodimer composed of the full‐length SLC30A5 protein. All results were visualized using ChimeraX and AlphaPickle. The putative zinc‐binding and manganese‐binding sites in SLC30A1 and SLC30A10 were predicted using the Metal Ion‐Binding Site Prediction and Docking Server (MIB).^[^ ^51^ ^]^ Root mean square deviation (RMSD) was calculated automatically using PyMOL after aligning the two structures.

#### 5.2.7 Generation of Stable Cell Lines Lacking SLC30A1 Expression

We used the p459 plasmid with two sgRNAs to generate HeLa cells lacking *SLC30A1* expression (SLC30A1‐KO). Vectors expressing the mutant forms of SLC30A1 were generated using the Mut Express II Fast Mutagenesis Kit V2 in the pTSB plasmid (TranSheepBio). The WT or mutant pTSB‐SLC30A1 vector, the psPAX2 packaging plasmid, and the pMD2.G envelope plasmid were then transfected into HEK293T cells to produce lentivirus. The lentivirus particles were collected in the cell culture medium and concentrated using lentivirus precipitation solution (#EMB810A‐1, ExCell Bio) in accordance with the manufacturer's instructions. The concentrated lentivirus particles were then applied to SLC30A1‐KO HeLa cells, and G418 was used to select for SLC30A1‐expressing cells 24 h after infection.

#### 5.2.8 Immunofluorescence

SLC30A1‐KO HeLa cells expressing either the WT or mutant versions of SLC30A1 were washed with PBS, fixed in 4% paraformaldehyde for 10 min, washed three times with PBS, and then permeabilized with 0.3% Triton X‐100 in PBS for 10 min. After blocking with 3% (w/v) BSA for 30 min, the cells were incubated with anti‐Flag antibody at 37 °C for 2 h. The cells were washed three times with PBS and then incubated with Cy3‐conjugated goat anti‐mouse IgG (#A0521, Beyotime) at 37 °C for 1 h. After washing three times with PBS, the nuclei were counterstained with DAPI (#D9542, Sigma‐Aldrich), and the cells were mounted using ProLong Diamond Antifade Mountant (#P36970, Thermo Fisher). Fluorescence images were obtained using a confocal microscope (Olympus).

#### 5.2.9 Zinc Cytotoxicity, Manganese Cytotoxicity, and Zinc Transport

SLC30A1‐KO HeLa cells were cultured in 96‐well plates and transfected with either WT or mutant SLC30A1. After 30 h, the cells were cultured for 24 h in excess ZnSO_4_ or MnCl_2_, after which the culture medium was replaced with Cell Counting Kit 8 working solution, and the cells were incubated for an additional 1 h at 37 °C. Absorbance at 450 nm was then measured using a microplate reader. To measure intracellular zinc levels, transfected cells were first incubated for 1 h with 2 µM FluoZin‐3 AM. After loading with FluoZin‐3 AM, the cells were washed once with PBS, and fluorescence was measured using a Varioskan Flash microplate reader (Thermo Fisher Scientific) with excitation and emission wavelengths of 494 nm and 516 nm, respectively. FluoZin‐3 fluorescence was measured at 1‐min intervals, and 20 µM ZnSO_4_ was applied after 4 min.

### 5.3 Statistical Analysis

Data were analyzed and plots were generated using GraphPad Prism version 9.0.0. Except where indicated otherwise, all summary data are presented as the mean ± SEM. Where indicated, groups were compared using the 2‐tailed unpaired Student's *t*‐test or one‐way or two‐way ANOVA, with Tukey's post hoc test. For the Kaplan‐Meier survival plots, analyzes were performed using the log‐rank (Mantel‐Cox) test. Differences with a *p*‐value < 0.05 were considered significant.

## Conflict of Interest

The authors declare no conflict of interest.

## Author Contributions

S.S., E.X., S.X., and S.J. contributed equally to this work. Conceptualization was carried out by FW, JM, SS, SX, and YZ; Methodology was applied by SS, SX, SJ, YZ, JM, and FW; Investigation was conducted by SS, EX, SX, SJ, SW, JS, RW, XS, YS, ZS, XW, JZ, ZC, XL, YZ, JM, and FW; Visualization was carried out by SS and SX; Funding acquisition was performed by FW, JM, SS, and SX; Project administration was carried out by FW, JM, SS, and SX; Supervision was dealt with by FW, JM, and YZ; Writing – original draft was by SS and SX; Writing – review & editing was conducted by all authors.

## Supporting information



Supporting Information

## Data Availability

The data that support the findings of this study are available from the corresponding author upon reasonable request.
