# Supplementary material for: The Intestinal Transporter SLC30A1 Plays a Critical Role in Regulating Systemic Zinc Homeostasis
Source: Adv Sci (Weinh). 2024 Oct 18;11(46):2406421. doi: 10.1002/advs.202406421 (PMC11633486; doi:10.1002/advs.202406421)
Supplement: Supplementary file 1 — Supporting Information [file ADVS-11-2406421-s001.pdf]

ADVANCED SCIENCE
Open Access

Supporting Information

for *Adv. Sci.*, DOI 10.1002/advs.202406421

The Intestinal Transporter SLC30A1 Plays a Critical Role in Regulating Systemic Zinc Homeostasis

Shumin Sun, Enjun Xie, Shan Xu, Suyu Ji, Shufen Wang, Jie Shen, Rong Wang, Xinyi Shen, Yunxing Su, Zijun Song, Xiaotian Wu, Jiahui Zhou, Zhaoxian Cai, Xiaopeng Li, Yan Zhang, Junxia Min* and Fudi Wang**

Supporting Information

Title: The Intestinal Transporter SLC30A1 Plays a Critical Role in Regulating Systemic Zinc Homeostasis

Shumin Sun, Enjun Xie, Shan Xu, Suyu Ji, Shufen Wang, Jie Shen, Rong Wang, Xinyi Shen, Yunxing Su, Zijun Song, Xiaotian Wu, Jiahui Zhou, Zhaoxian Cai, Xiaopeng Li, Yan Zhang, Junxia Min*, Fudi Wang**

This file includes:

Supplemental Figure 1-15

Supplemental Table 1-3

Supplemental figures

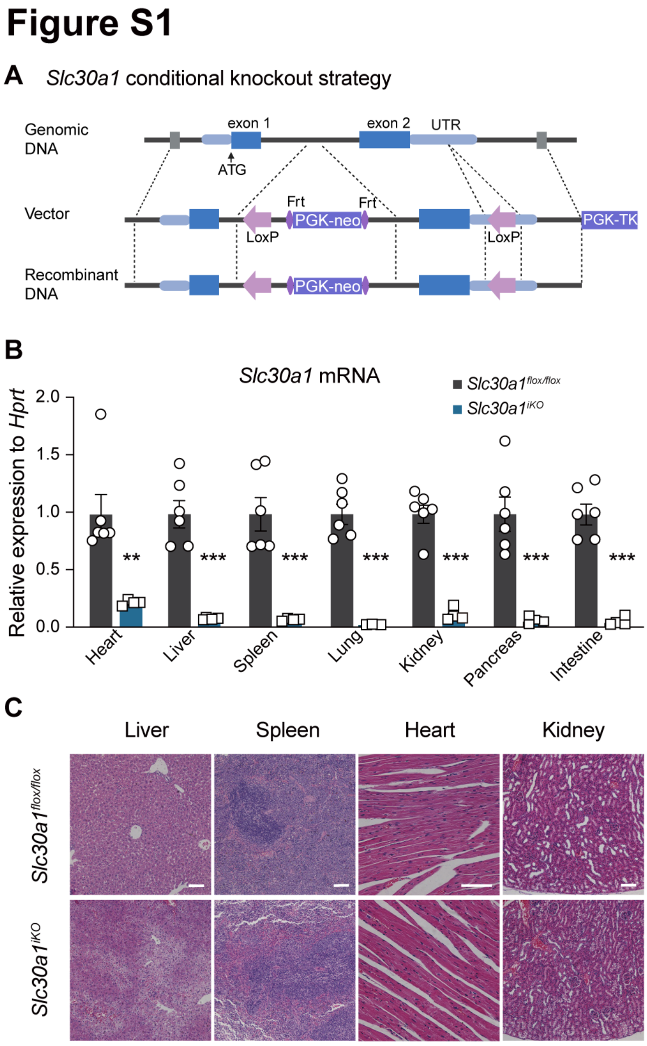

Figure S1. Characterization of inducible global *Slc30a1* knockout mice, related to Figure 1. (A) Strategy for generating *Slc30a1*$^{flox/flox}$ mice. (B) Summary of *Slc30a1* mRNA measured in the indicated tissues obtained from *Slc30a1*$^{flox/flox}$ and *Slc30a1*^{iKO} mice 6 days after tamoxifen induction (dpi6). (C) Representative images of the indicated tissue sections obtained from *Slc30a1*$^{flox/flox}$ and *Slc30a1*^{iKO} mice at dpi6 and stained with hematoxylin and eosin (H&E). In this and subsequent figures, results are representative of at least two independent experiments; in addition, unless indicated otherwise all summary data are presented as the mean ± SEM are plotted, and each data point represents one mouse. $**p < 0.01$, and $***p < 0.001$ (Student's *t*-test). Scale bars, 100 μm.

Figure S2

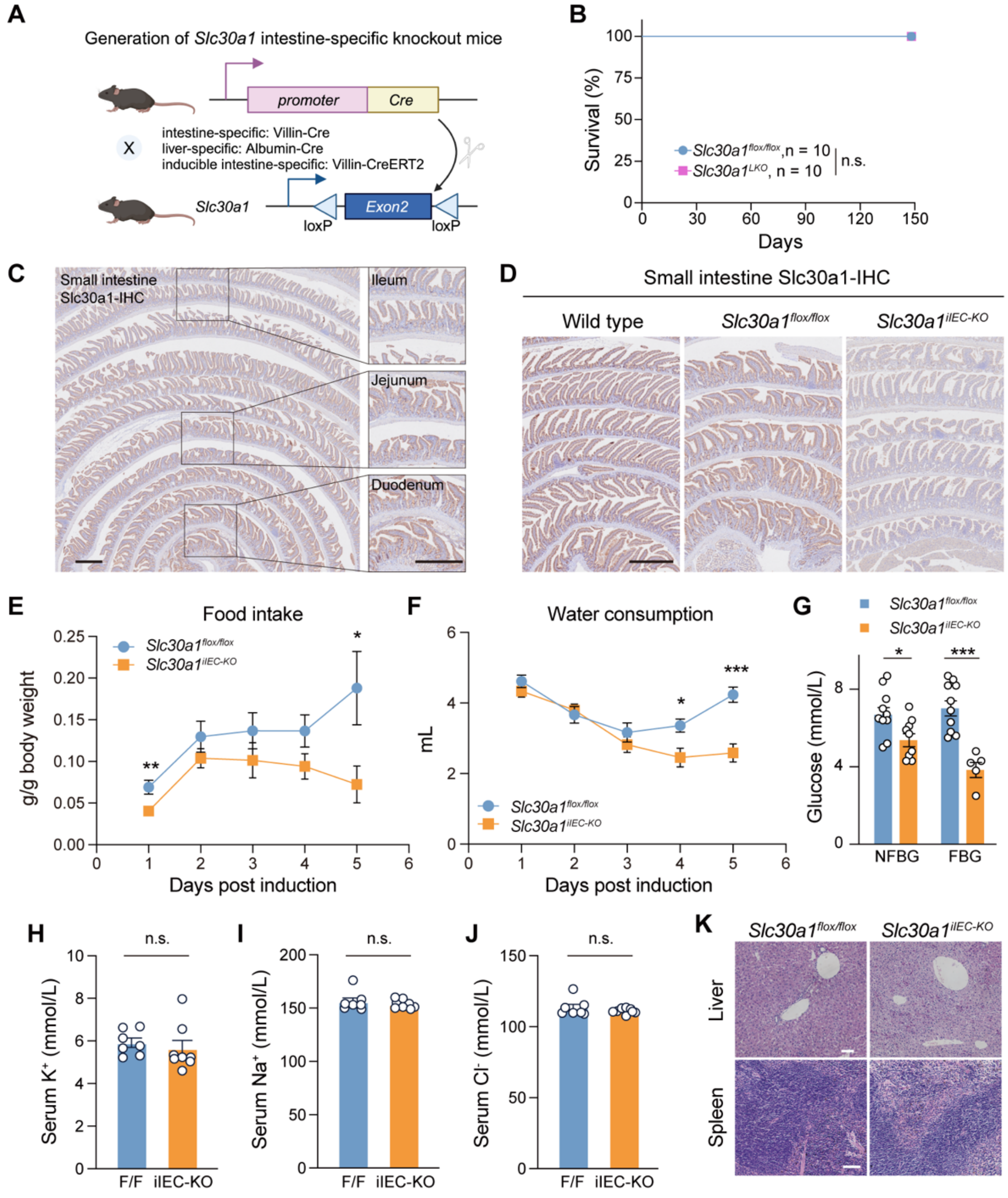

Figure S2. Characterization of intestine-specific *Slc30a1* knockout mice, related to Figure 1. (A) Strategy for generating the tissue-specific *Slc30a1* conditional knockout mice. (B) Survival curve of the indicated mice. (C) Representative images of Swiss rolls prepared from the small intestine samples obtained from C57BL/6 wild type mice and stained with anti-Slc30a1 antibody. (D) Representative images of Swiss rolls prepared from the small intestine samples obtained from wild type, *Slc30a1*$^{flox/flox}$, and *Slc30a1*$^{iIEC\text{-}KO}$ mice, and stained with anti-Slc30a1 antibody. (E and F) Time course of food intake (E) and water consumption (F) following tamoxifen induction in *Slc30a1*$^{flox/flox}$ and *Slc30a1*$^{iIEC\text{-}KO}$ mice (n = 14 mice per group). (G–J) Summary of non-

fasting blood glucose (NFBG) and fasting blood glucose (FBG) levels (G), and serum K^+ (H), Na^+ (I), and Cl^- (J) levels measured in *Slc30a1^flox/flox^* and *Slc30a1^iIEC-KO^* mice at dpi6. (K) Representative images of liver and spleen sections obtained from *Slc30a1^flox/flox^* and *Slc30a1^iIEC-KO^* mice at dpi6 and stained with H&E. *$p < 0.05$, **$p < 0.01$, ***$p < 0.001$, and n.s., not significant (Student's *t*-test (C–H) or log-rank (Mantel-Cox) test (B)). Scale bars, 500μm (C and D) and 100 μm (K).

Figure S3

A

Variant information

Phenotype code	Phenotype description	Variant	Annotation
20002_1462	Non-cancer illness, self-reported: crohns disease	1:211740959:C:T	Modifier

Sample information

Sex	Controls	Cases	p-value
Female	n=193,528	n=628	0.471807
Male	n=166,517	n=471	9.39×10^{-5}

B

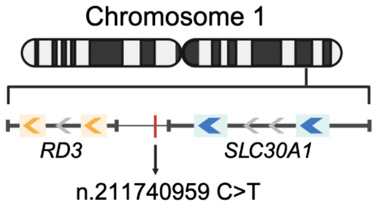

Figure S3. Analysis of a *SLC30A1* variant in patients with Crohn's disease, related to Figure 1. (A) Information of a *SLC30A1* variant found in patients with Crohn's disease, identified by analyzing the data obtained from UK Biobank. (B) Schematic diagram showing the intergenic modifier mutation between the *RD3* and *SLC30A1* genes on chromosome 1 (n.211740959 C>T) in male patients with Crohn's disease.

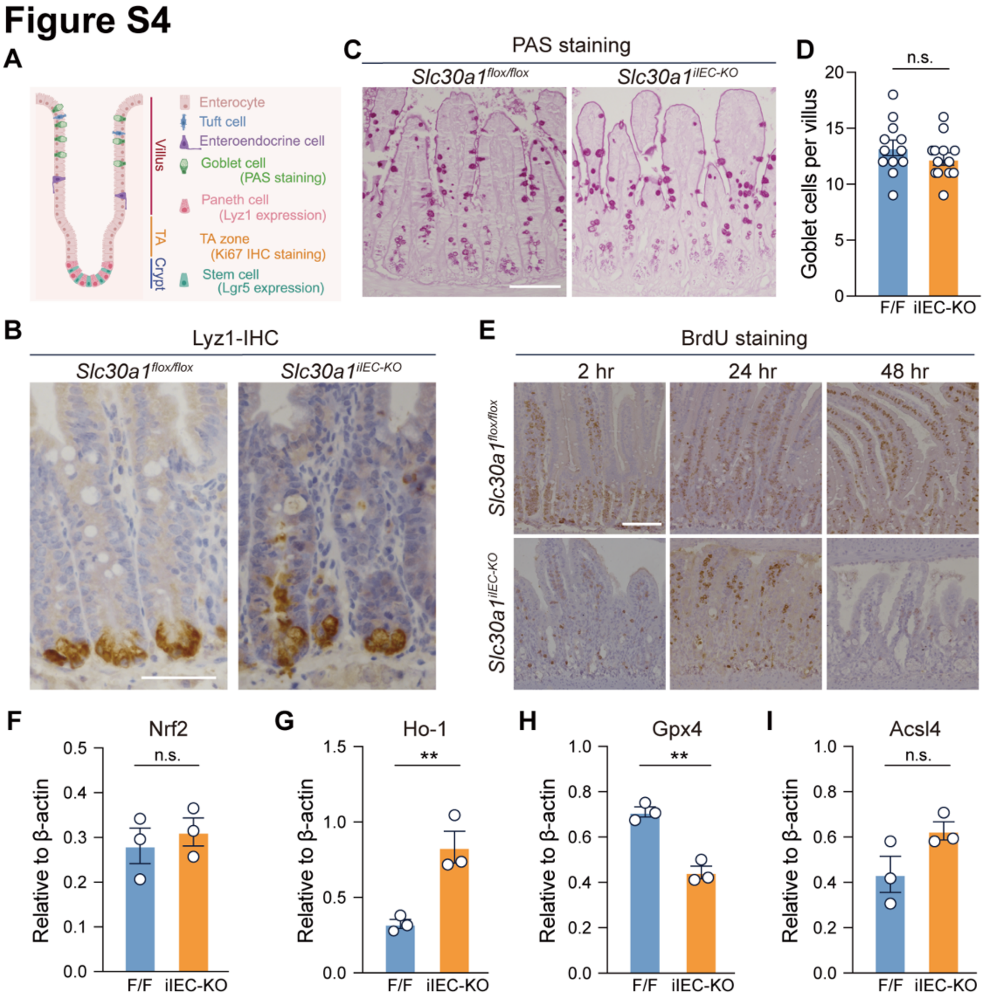

Figure S4. Analysis of various types of intestinal epithelial cells in intestine-specific *Slc30a1* knockout mice, related to Figure 2. (A) Diagram depicting the intestinal structure and cell types in villi and crypts; also shown are TA (transit amplifying) cells. (B) Representative images of small intestine sections obtained from *Slc30a1*$^{flox/flox}$ and *Slc30a1*$^{iIEC\text{-}KO}$ mice at dpi6 and stained for lysozyme 1 (Lyz1) to label Paneth cells. (C and D) Representative images of small intestine sections obtained from *Slc30a1*$^{flox/flox}$ and *Slc30a1*$^{iIEC\text{-}KO}$ mice at dpi6 and stained with PAS to label goblet cells (C), and summary of PAS-positive cells per villus (D) (n = 12 villi per group pooled from 3 biological replicates). (E) Representative images of small intestine sections prepared from *Slc30a1*$^{flox/flox}$ and *Slc30a1*$^{iIEC\text{-}KO}$ mice at dpi6 2, 24, and 48 hours after an i.p. injection of BrdU. (F–I) Summary of Nrf2 (F), Ho-1 (G), Gpx4 (H), and Acsl4 (I) proteins in IECs obtained from the indicated mice at dpi6, measured using western blot analysis (related to Figure 2M). $^{**}p < 0.01$ and n.s., not significant (Student's *t*-test). Scale bars, 50 µm (B) and 100 µm (C and E).

Figure S5

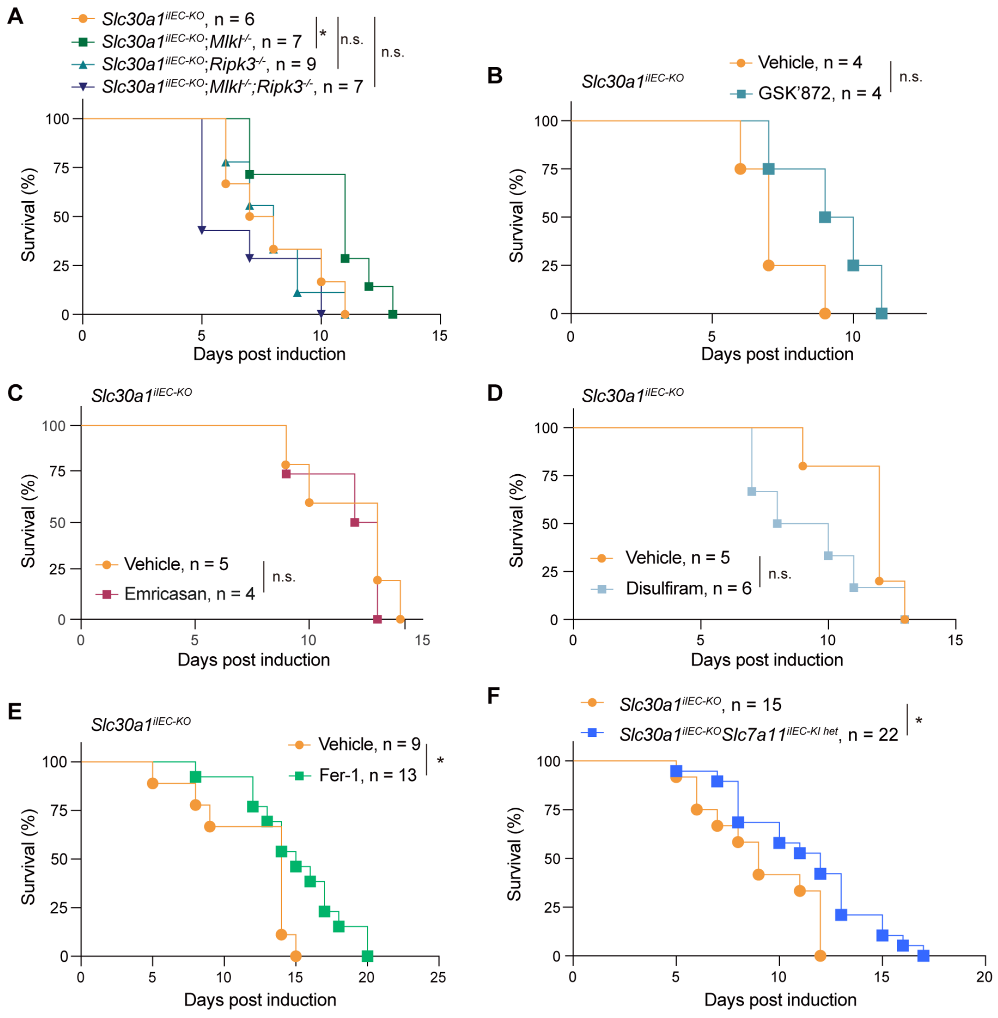

Figure S5. Survival curves of *Slc30a1$^{iIEC\text{-}KO}$* mice with various genetic or pharmacological interventions, related to Figure 2. (A) Survival curve of the indicated mice following tamoxifen induction. (B–E) Survival curves of *Slc30a1$^{iIEC\text{-}KO}$* mice treated with a necroptosis inhibitor (B), apoptosis inhibitor (C), pyroptosis inhibitor (D), or ferroptosis inhibitor (E). (F) Survival curve of *Slc30a1$^{iIEC\text{-}KO}$* mice and *Slc30a1$^{iIEC\text{-}KO}$* mice overexpressing *Slc7a11* in the intestinal cells. *$p < 0.05$ and n.s., not significant (log-rank (Mantel-Cox) test).

Figure S6

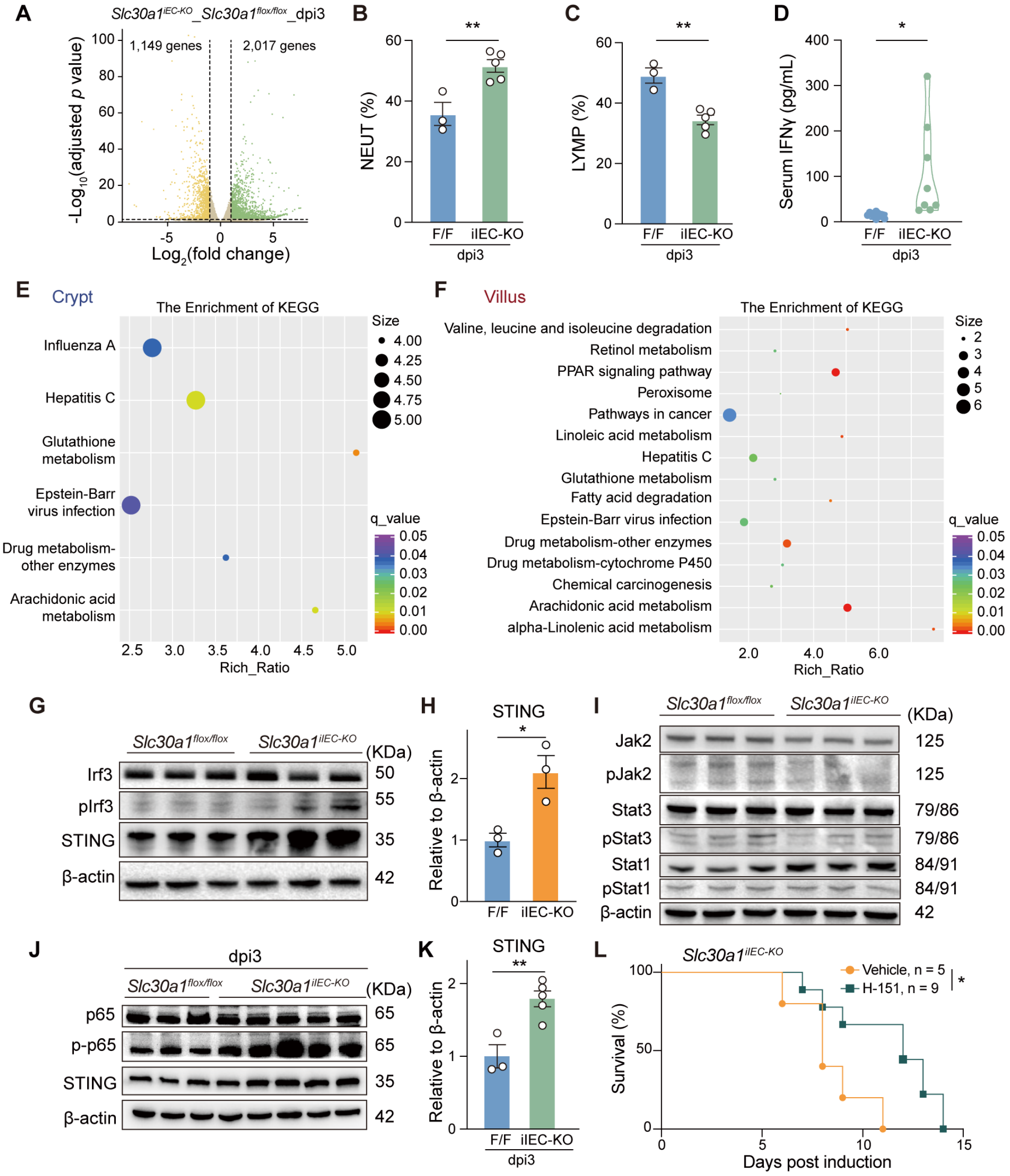

Figure S6. Loss of intestinal Slc30a1 induces a characteristic inflammatory response at dpi3 and dpi6, related to Figure 3. (A) Volcano plot of differentially expressed genes identified using RNA-seq analysis of IECs isolated from the small intestine of *Slc30a1*$^{flox/flox}$ and *Slc30a1*$^{iIEC-KO}$ mice at dpi3 (n = 3 biological replicates). Genes with $|\log_2(\text{fold change})| \geq 1$ and an adjusted *p*-value of <0.05 were considered significant. Each dot represents a gene, with green and yellow dots representing genes that were significantly upregulated and downregulated, respectively. (B–D) Summary of the percentage of neutrophils (B), the percentage of lymphocytes (C), and serum interferon gamma (IFNγ) levels (D) measured in the indicated mice at dpi3. (E and F) Significantly altered pathways based on KEGG enrichment analysis of differentially

expressed genes in the crypts (E) and villi (F) of *Slc30a1*$^{iIEC\text{-}KO}$ mice at dpi6 (n = 2–3 biological replicates). (G–I) Western blot analysis of the indicated proteins related to interferon regulation (G and I) and summary of STING protein levels (H) in IECs isolated from the indicated mice at dpi6; β-actin was included as a loading control. (J and K) Western blot analysis of p65, phosphorylated p65 (p-p65), and STING proteins (J), and summary of STING protein levels (K) in IECs isolated from the indicated mice at dpi3; β-actin was included as a loading control. (L) Survival curve of *Slc30a1*$^{iIEC\text{-}KO}$ mice treated with the STING inhibitor H-151 or vehicle. $^*p < 0.05$ and $^{**}p < 0.01$ (Student's *t*-test (B–D, H, and K) or log-rank (Mantel-Cox) test (L)).

Figure S7

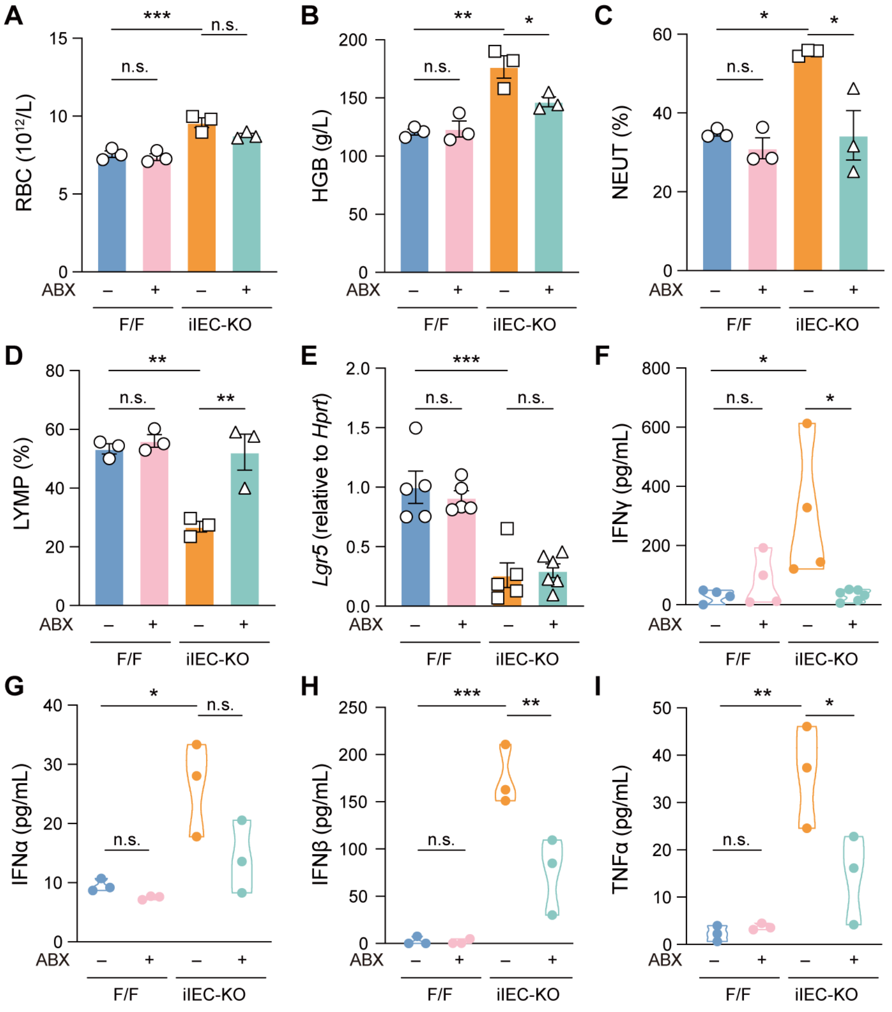

Figure S7. Treating intestine-specific *Slc30a1* knockout mice with an antibiotic cocktail prevents overactivation of the inflammatory response, related to Figure 3. (A–D) Summary of the RBC count (A), hemoglobin concentration (B), percentage of neutrophils (C), and percentage of lymphocytes (D) in blood samples obtained from *Slc30a1*$^{flox/flox}$ and *Slc30a1*$^{iIEC\text{-}KO}$ mice at dpi6. (E) Summary of *Lgr5* mRNA measured in IECs obtained from the indicated mice at dpi6. (F–I) Summary of the indicated cytokines measured in the serum of the indicated mice at dpi6. **p* < 0.05, ***p* < 0.01, ****p* < 0.001, and n.s., not significant (two-way ANOVA with Tukey's post hoc test).

Figure S8

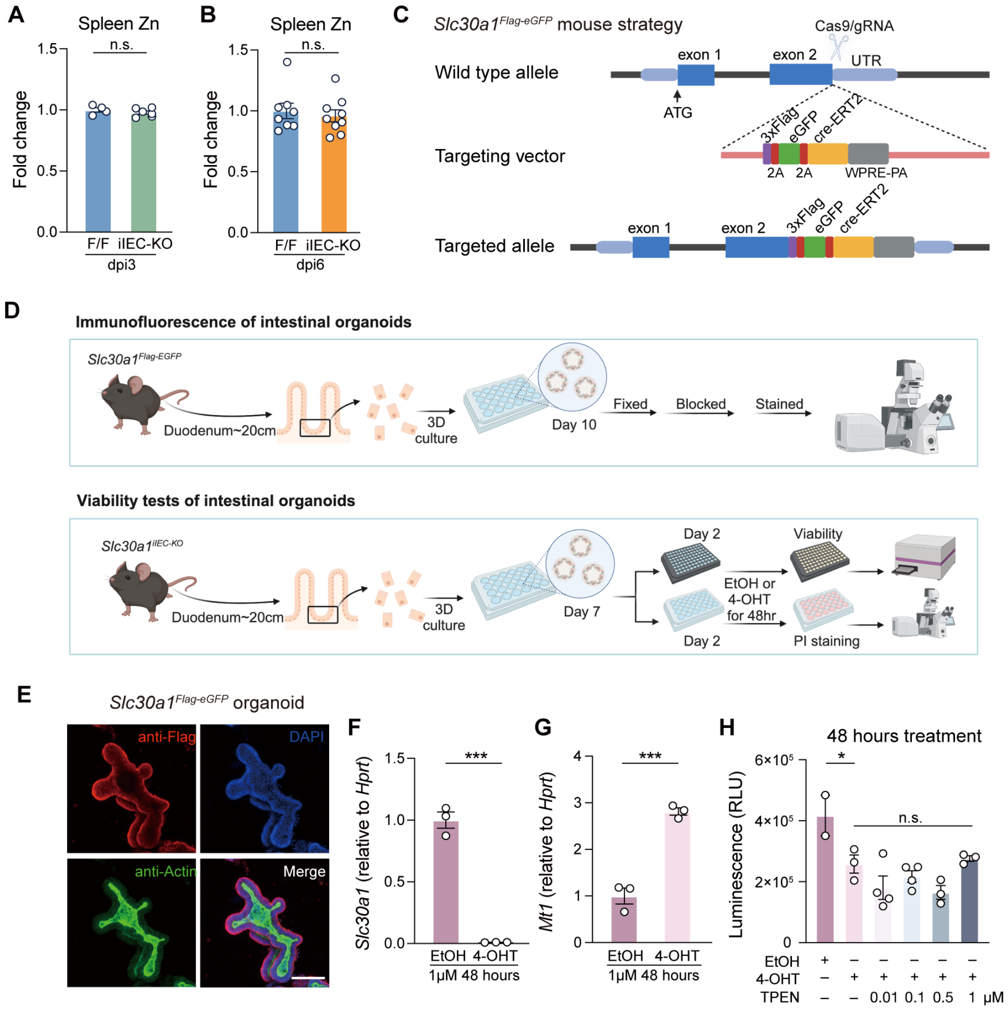

Figure S8. Dysregulation of systemic zinc underlies the death of *Slc30a1$^{iIEC\text{-}KO}$* mice, related to Figure 4. (A and B) Summary of zinc levels in the spleen of the indicated mice at dpi3 (A) and dpi6 (B) measured using ICP-MS. (C) Strategy for generating *Slc30a1$^{Flag\text{-}eGFP}$* mice. (D) Diagram depicting the experimental processes of intestinal organoids. (E) *Z*-stacked fluorescence images of organoids derived from *Slc30a1$^{Flag\text{-}eGFP}$* mice and immunostained with anti-Flag (red) and anti-Actin (green) antibodies; the nuclei were counterstained with DAPI (blue). (F and G) Summary of *Slc30a1* (F) and *Mt1* (G) mRNA measured in organoids derived from *Slc30a1$^{iIEC\text{-}KO}$* mice and treated for 48 hours with ethanol (EtOH) or 1 μM 4-hydroxytamoxifen (4-OHT) (n = 3 biological replicates per group). (H) Summary of cell viability measured in organoids derived from *Slc30a1$^{iIEC\text{-}KO}$* mice and treated for 48 hours with EtOH or 1 μM 4-OHT in the absence or presence of the indicated concentrations of the zinc chelator TPEN (n = 2–4 biological replicates per group). *$p < 0.05$, ***$p < 0.001$, and n.s., not significant (Student's *t*-test (A, B, F, and G) or one-way ANOVA with Tukey's post hoc test (H)).

Scale bars, 100 μm.

Figure S9

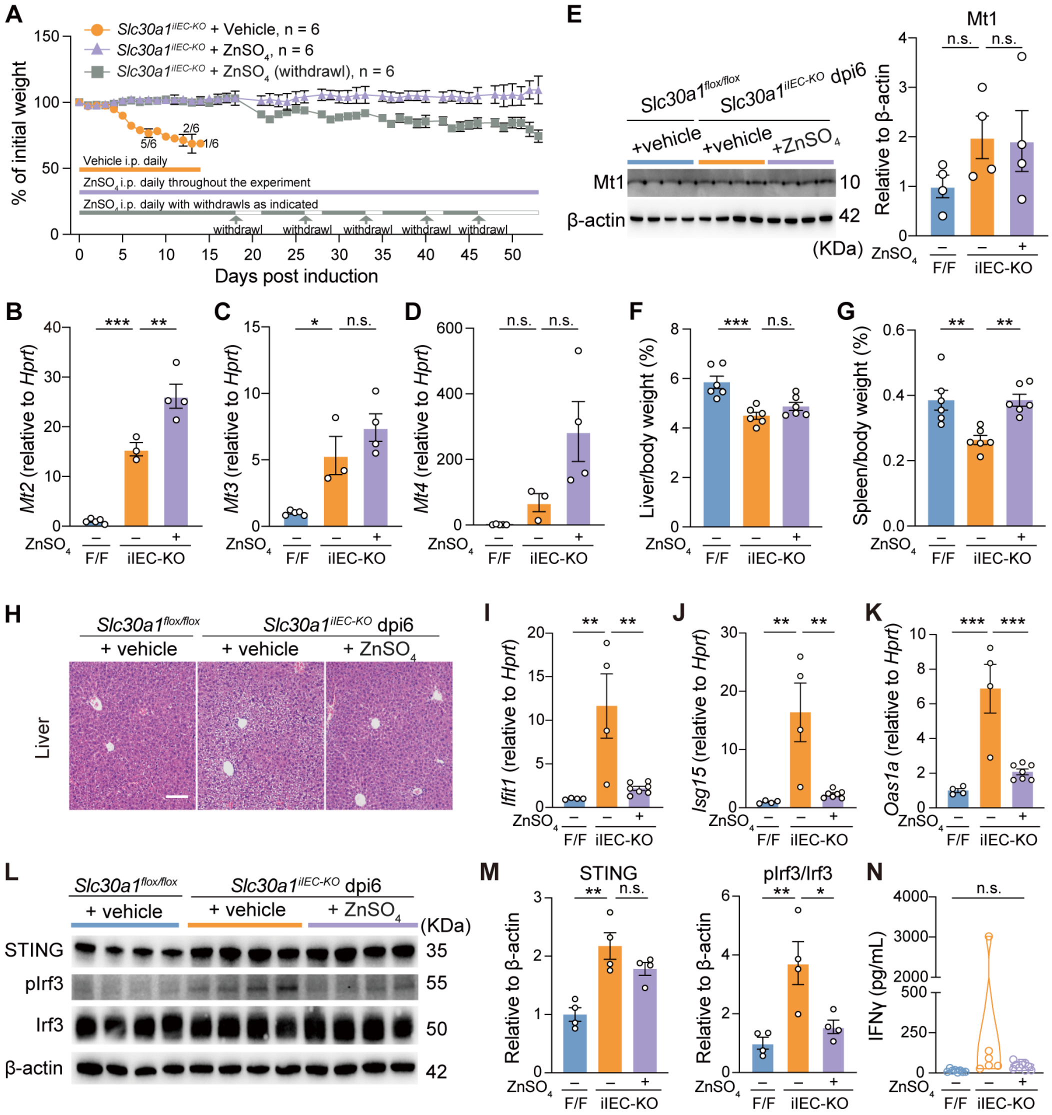

Figure S9. Intraperitoneal injections of zinc ameliorate the phenotype in intestine-specific *Slc30a1* knockout mice, related to Figure 4. (A) Time course of relative body weight measured in *Slc30a1*$^{iIEC\text{-}KO}$ mice receiving zinc supplementation or vehicle (saline). (B–D) Summary of *Mt2* (B), *Mt3* (C), and *Mt4* (D) mRNA measured in IECs isolated from the indicated mice at dpi6. (E) Western blot analysis of Mt1 protein levels in IECs isolated from the indicated mice at dpi6; β-actin was included as a loading control. (F and G) Summary of liver (F) and spleen (G) weight measured at dpi6 in *Slc30a1*$^{flox/flox}$ and *Slc30a1*$^{iIEC\text{-}KO}$ mice. (H) Representative images of liver sections obtained from the indicated mice at dpi6 and stained with H&E. (I–K) Summary of the indicated mRNA levels measured in IECs isolated from the indicated mice at dpi6. (L and M) Western blot analysis (L) and summary (M) of Irf3, phosphorylated Irf3 (pIrf3), and STING proteins measured in IECs isolated from the indicated mice at dpi6; β-actin

was included as a loading control. (N) Summary of interferon gamma (IFNγ) measured in the serum of the indicated mice at dpi6. *$p < 0.05$, **$p < 0.01$, ***$p < 0.001$, and n.s., not significant (one-way ANOVA with Tukey's post hoc test). Scale bar, 100 μm.

Figure S10

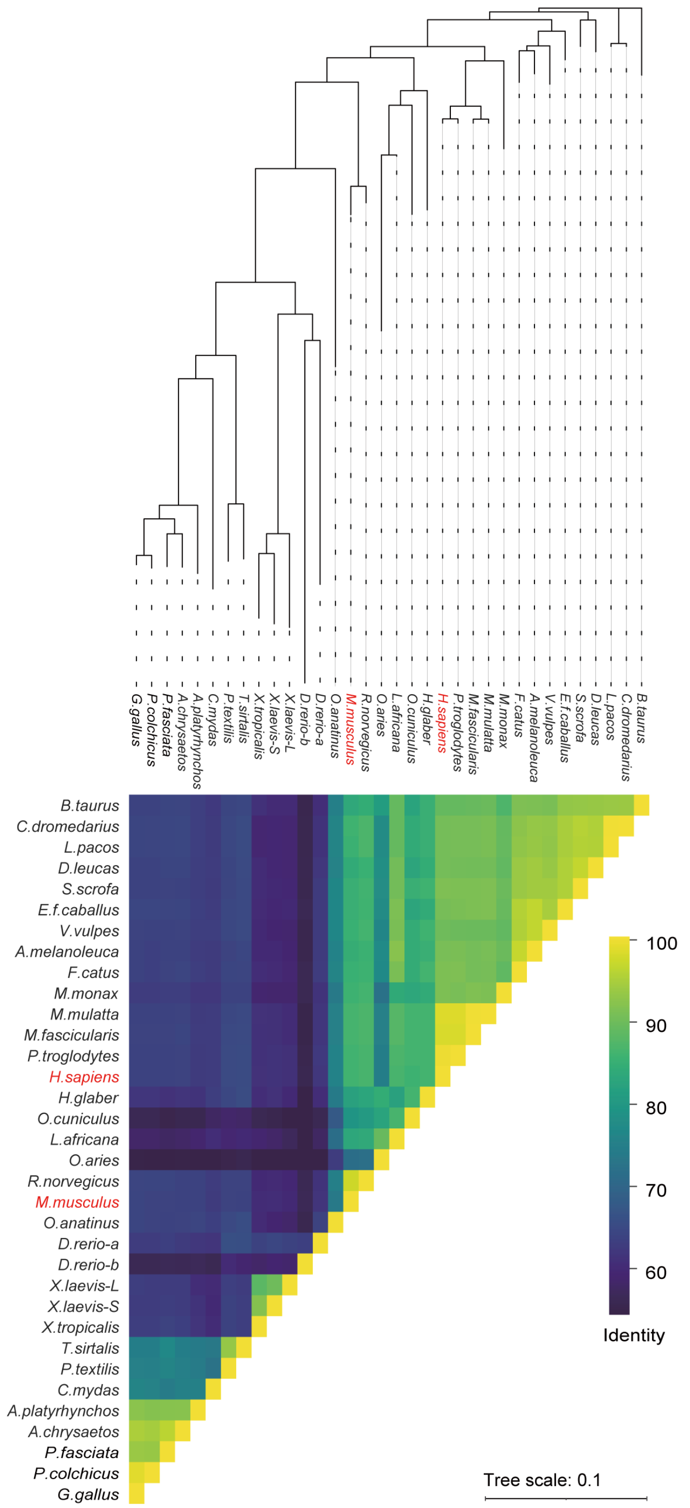

Figure S10. Phylogenetic tree of SLC30A1 orthologs, related to Figure 5. Phylogenetic tree depicting the SLC30A1 orthologs in the indicated vertebrate species. The heat map indicates identity at the amino acid level.

Figure S11

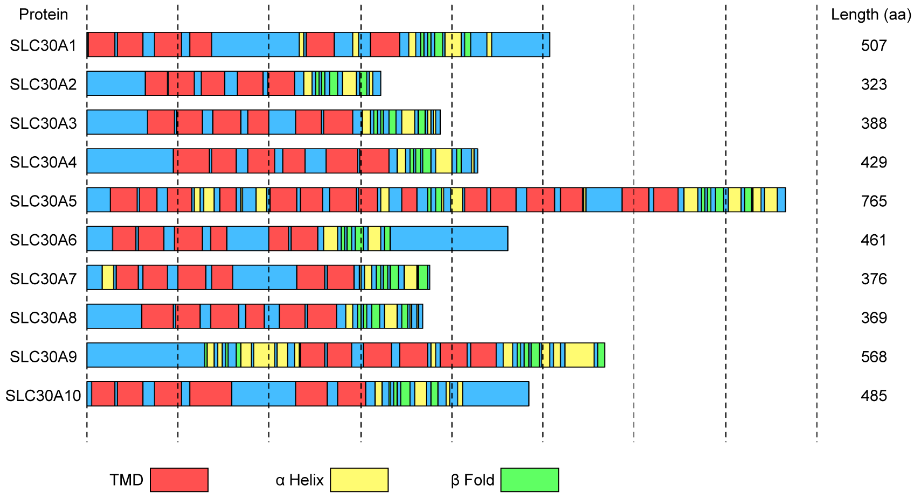

Figure S11. Predicted secondary structures of all 10 SLC30A family members, related to Figure 5. The secondary structure of human SLC30A1 through SLC30A10 is shown, based on the predictions obtained using AlphaFold2-Multimer. The length of each protein is shown, and the TMDs (transmembrane domains), α-helices, and α-folds are indicated in red, yellow, and green, respectively.

Figure S12

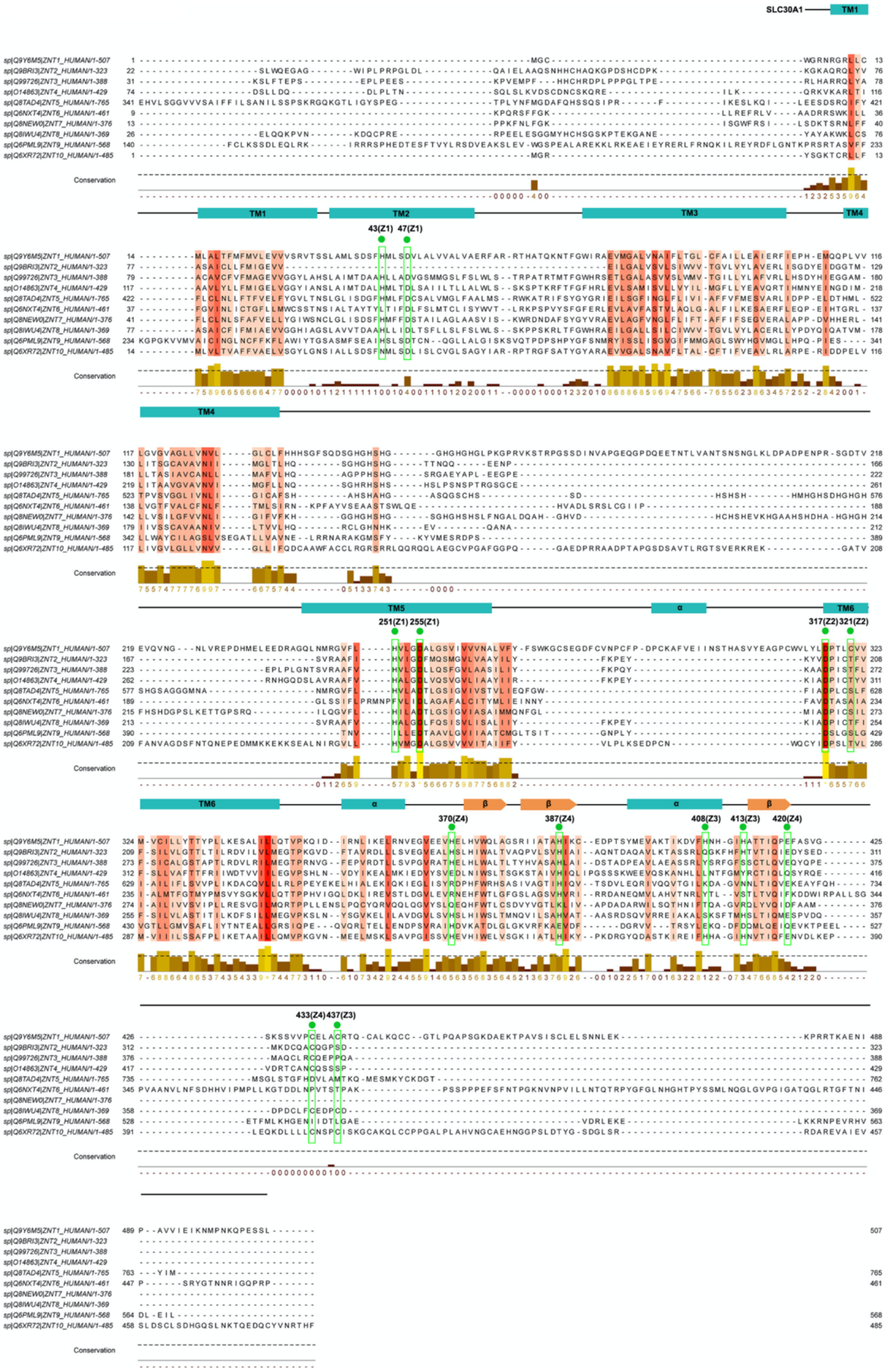

Figure S12. Amino acid alignment of human SLC30A1 through SLC30A10, related to Figure 5. The zinc-binding sites in SLC30A1 are indicated with green dots,

and the various domains in SLC30A1 are shown in blue and orange shading. The level of conservation for each residue is shown below the sequences.

Figure S13

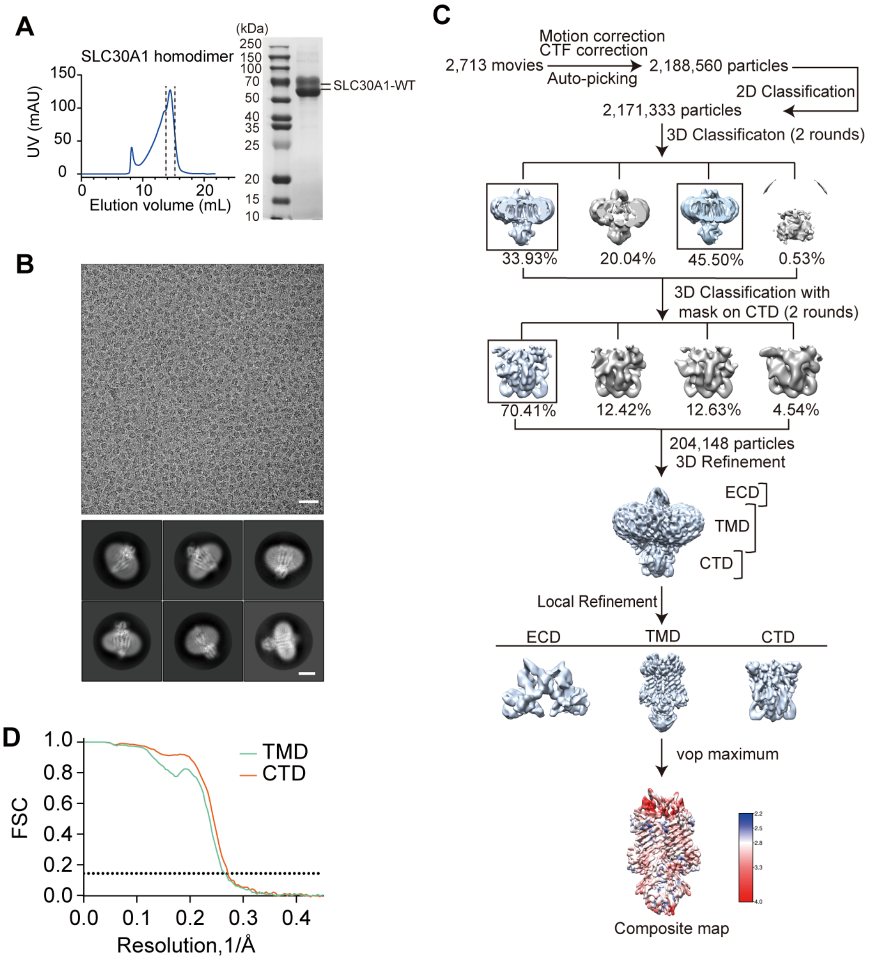

Figure S13. Purification, cryo-EM data processing, and analysis of the electron density map of human SLC30A1, related to Figure 5. (A) Representative elution profile of WT SLC30A1 homodimers (left) and SDS-PAGE analysis of the size-exclusion chromatography peak (right). (B) Cryo-EM micrograph (upper panel, scale bar = 30 nm) and reference-free two-dimensional class averages (lower panel, scale bar = 5 nm) of WT SLC30A1. A representative cryo-EM micrograph obtained from 2,713 movies and 6 representative two-dimensional class averages determined using approximately 2 million particles are shown. (C) Flow chart depicting the steps used to process the cryo-EM data obtained for SLC30A1. (D) Gold-standard Fourier shell correlation (FSC) curves of local refined TMD and CTD domains in SLC30A1.

Figure S14

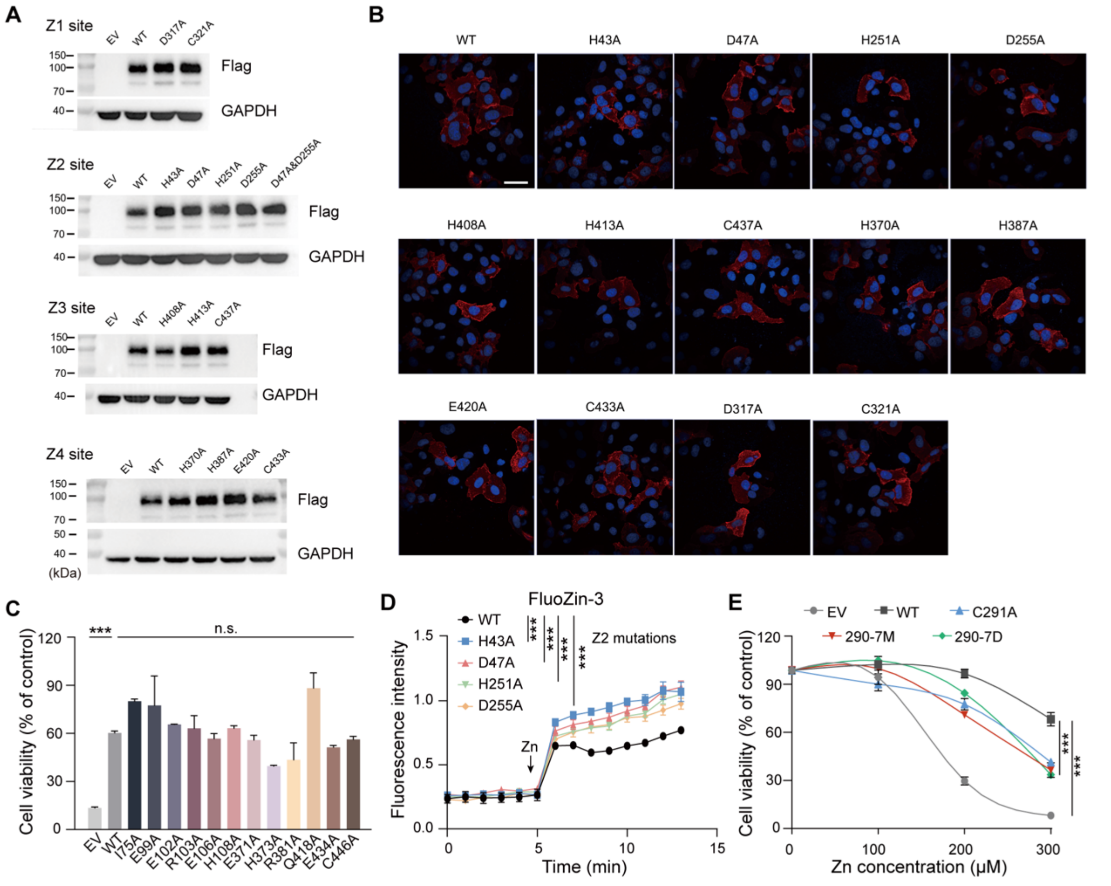

Figure S14. Expression and subcellular location of the WT and mutant versions of SLC30A1, related to Figure 5. (A) SLC30A1-KO HeLa cells transfected with an empty vector (EV), a vector expressing WT SLC30A1, or a vector expressing SLC30A1 with the indicated mutations in the putative zinc-bindings sites were lysed, and western blot analysis was performed using an anti-Flag antibody. GAPDH was included as a loading control. (B) SLC30A1-KO HeLa cells expressing WT SLC30A1 or the indicated SLC30A1 mutants were immunostained for SLC30A1 (red); the nuclei were counterstained with DAPI (blue). The scale bar represents 40 μm. (C) SLC30A1-KO HeLa cells expressing the indicated constructs were treated with high zinc, and cell viability was measured and is plotted relative to control (untreated) cells. (D) Cells expressing either WT SLC30A1 or SLC30A1 with the indicated mutations in the Z2 site were loaded with the fluorescent zinc indicator FluoZin-3 AM. Where indicated (arrow), high zinc was applied to the cells (n = 3 biological replicates per group). (E) Cell viability was measured in SLC30A1-KO HeLa cells expressing the indicated constructs and treated with the indicated concentrations of zinc. ***$p < 0.001$, and n.s., not significant (one-way ANOVA with Tukey's post hoc test).

Figure S15

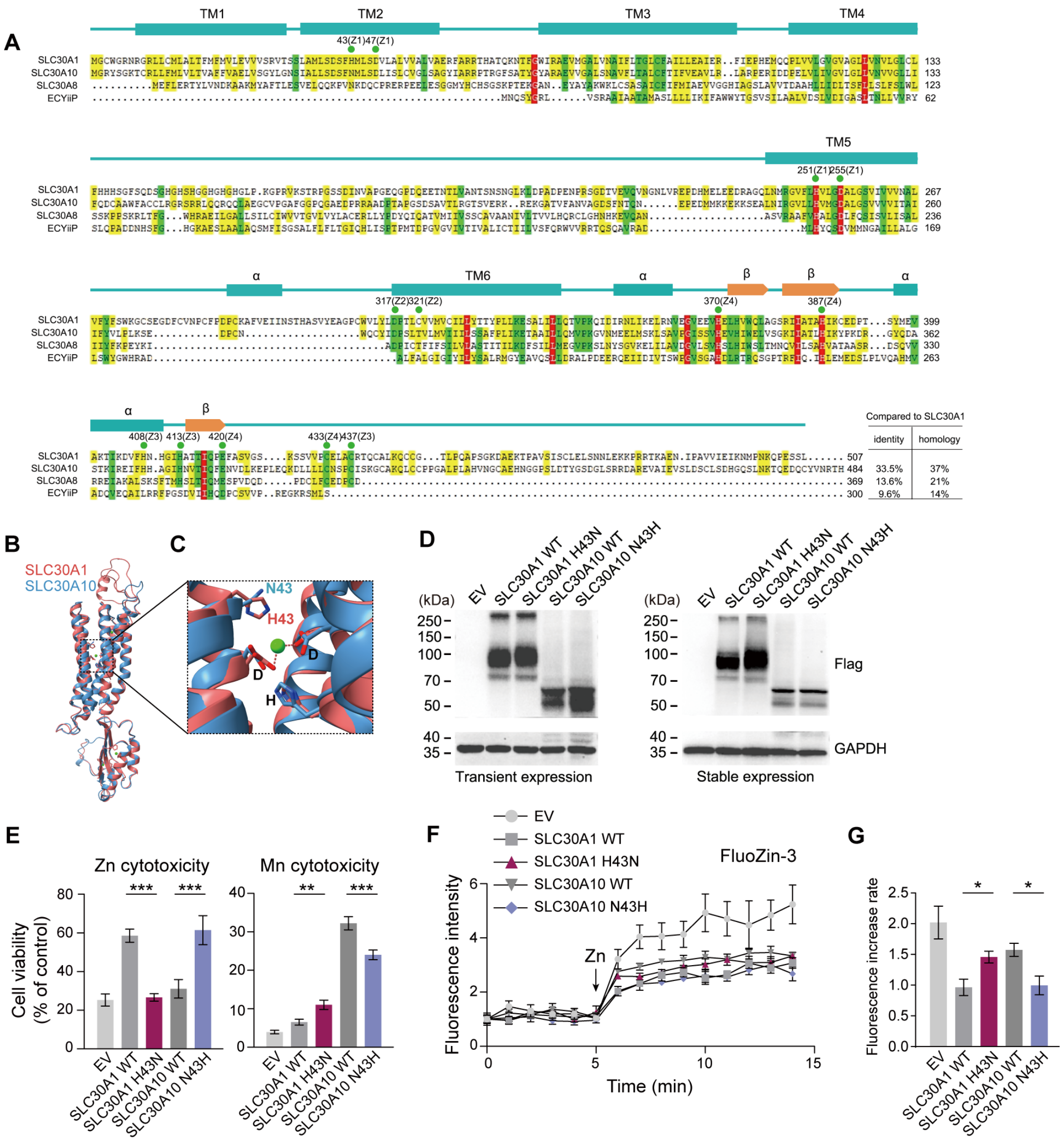

Figure S15. Structural analysis of the transport selectivity of SLC30A1, related to Figure 5. (A) Amino acid alignment of human SLC30A1, human SLC30A10, human SLC30A8, and *E. coli* YiiP (ECYiiP) generated using DNAMAN. The putative zinc-binding sites and domains in SLC30A1 are indicated. (B) Structural alignment of an SLC30A1 monomer and an SLC30A10 monomer. (C) Enlarged view of the site in SLC30A1 and SLC30A10 that plays a predominant role in determining the transporter's ion selectivity, with key residues indicated. (D) Cells expressing the indicated constructs were lysed, followed by western blot analysis using an anti-Flag antibody. GAPDH was included as a loading control. (E) Summary of the viability of SLC30A1-KO HeLa cells expressing the indicated SLC30A1 or SLC30A10 constructs after incubation for 24 hours in 100 μM $ZnSO_4$ or 200 μM $MnCl_2$ (n = 3 biological

replicates per group). (F) Time course of FluoZin-3 fluorescence measured in cells expressing the indicated constructs; where indicated, high zinc was applied to the cells (n = 3 biological replicates for each group). (G) Summary of the initial rate of increase in FluoZin-3 fluorescence in the indicated cells upon the addition of zinc (n = 3 biological replicates per group). *$p < 0.05$, **$p < 0.01$, and ***$p < 0.001$ (one-way ANOVA with Tukey's post hoc test).

Table S1. Identification of the SLC30A1 protein bands in SDS-PAGE using mass spectroscopy.

Sample L

Gene names	Unique peptides	Sequence coverage [%]	Sequence length	Intensity
SLC30A1	22	53.5	507	154760000000
HSPA1A;HSPA1B;HEL-S-103	18	65.4	641	54465000000
PCDHB14	1	0.8	798	18219000000
HSPA6;HSPA7	1	11.7	643	4550600000
HSPA8;HEL-S-72p	8	24.8	646	2477600000
LRRC14B	1	1.4	514	1530300000
PCCA	16	25.1	728	1228900000
UBC;UBB;DKFZp434K0435;UbC;HEL112;UBA52;RPS27A	2	32.8	685	937500000
GAPDH;GAPD;HEL-S-162eP	1	4.2	335	696660000
HSPA9;HEL-S-124m	10	19.1	679	583220000

Sample S

Gene names	Unique peptides	Sequence coverage [%]	Sequence length	Intensity
SLC30A1	22	54.4	507	152570000000
HSPA1A;HEL-S-103;HSPA1B	3	15.3	641	1057500000
GAPDH;GAPD;HEL-S-162eP	1	4.2	335	427110000
ALB	8	14.4	609	320620000
HSPA1L	0	10.3	641	305160000
PCCB;DKFZp451E113	9	23.4	539	294060000
MROH1	1	1.3	1641	264350000
UBC;UBB;DKFZp434K0435;UbC;HEL112;UBA52;RPS27A	2	32.8	685	182080000
POTEE;ACTB;ACTG1;PS1TP5BP1;POTEF;ACTA1;POTEKP;POTEI;ACTC1;ACT;POTEM;ACTA2;ACTG2;POTEJ	4	5.2	1075	167780000
HIST1H4A;HIST1H4H	2	21.4	103	155170000

Table S2. Cryo-EM data collection, model refinement, and validation statistics

	SLC30A1-WT
**Data collection and processing**	
Magnification	49,310
Voltage (kV)	300
Electron exposure ($e^{-}/Å^2$)	64
Defocus range (μm)	-1.0 ~ -2.5
Pixel size (Å)	1.014
Symmetry imposed	C2
Initial particle projections (no.)	2,188,560
Final particle projections (no.)	204,148
Map resolution (Å)	3.7
FSC threshold	0.143
Map resolution range (Å)	2.2-4.0
**Refinement**	
Initial model used	ZnT8
Model resolution (Å)	3.7
FSC threshold	0.143
Model resolution range (Å)	2.2-4.0
Map sharpening *B* factor ($Å^2$)	-98.60
Model composition	
Non-hydrogen atoms	4,910
Protein residues	658
B factors ($Å^2$)	
Protein	70.16
Ligand	113.21
R.m.s. deviations	
Bond lengths (Å)	0.003
Bond angles (°)	0.666
Validation	
MolProbity score	2.05
Clash score	7.48
Rotamer outliers (%)	2.35
Ramachandran plot	
Favored (%)	94.77
Allowed (%)	5.23
Disallowed (%)	0.00

Table S3. Primer sequences used for quantitative PCR and genotyping

Genes	Forward (5' → 3')	Reverse (5' → 3')
**For quantitative PCR**		
Hmox1	AAGCCGAGAATGCTGAGTTCA	GCCGTGTAGATATGGTACAAGGA
Hprt	AAGCTTGCTGGTGAAAAGGA	TTGCGCTCATCTTAGGCTTT
Ifit1	GCCTATCGCCAAGATTTAGATGA	TTCTGGATTTAACCGGACAGC
Irf7	CCAGCTCTCACCGAGCG	CGGCCCTTGTACATGATGGT
Isg15	AGTGATGCTAGTGGTACAGAACT	CAGTCTGCGTCAGAAAGACCT
Lgr5	TCTCCTACATCGCCTCTGCT	GTGAACGCTCCCTTGGGAAT
Mt1	CCTGCAAGAACTGCAAGTG	TGGGCACATTTGGAGCA
Mt2	GCCTGCAAATGCAAACAATGC	AGCTGCACTTGTCGGAAGC
Mt3	ACCTGCCCCTGTCCTACTG	CCTTGGCACACTTCTCACATC
Mt4	TAAAACCTGTCGTAAAAGCTGCT	ACTTGTCTGAACCCCCTTTGC
Oas1a	CCTTTGATGTCCTGGGTCATGT	TTCTGGGATCAGGCTTGCTG
Slc30a1	GGTCGTGAATGCCTTGGTCT	TCAGGAAAACACGGGTTCGT
**For genotyping**		
Slc30a1 Flox	CAGAGAGGTGACGCAGTGTT	AAGGCATTCACGACCACGAT
Ubc Cre^{ERT}	GACGTCACCCGTTCTGTT G	AGGCAAATTTTGGTGTACGG
Villin Cre	CAAGCCTGGCTCGACGGCC	CGCGAACATCTTCAGGTTCT
Villin Cre^{ERT2}	CGCGAACATCTTCAGGTTCT	CAAGCCTGGCTCGACGGCC
Ripk3 KO	ACATGCATGGTCATGCACACACAT	GTCGAGGGACCTAATAACTTCGTA
Fadd KO	GAAGCGCATGCGTCTGATT	GCGCTGCAGTAGATCGTGT
Mlkl KO	CAGCACAAATCCCATCCACTC	TAAACCTGAAGCAGCAGCAAC
Slc7a11 KI	TCATGTCTGGATCCCCAT CAAGC	CTCCAGCTGACACTCGTGCT
